# Molecular Alchemy: Converting Stress into Resilience via Secondary Metabolites and Calcium Signaling in Rice

**DOI:** 10.1186/s12284-025-00783-7

**Published:** 2025-05-05

**Authors:** Muhammad Ikram, Maria Batool, Maaz Ullah, Burhan Khalid, Ali Mahmoud El-Badri, Ibrahim A. A. Mohamed, Lei Zhang, Jie Kuai, Zhenghua Xu, Jie Zhao, Jing Wang, Bo Wang, Guangsheng Zhou, Haseeb Ur Rehman

**Affiliations:** 1 MOA Key Laboratory of Crop Ecophysiology and Farming System in the Middle Reaches of the Yangtze River, College of Plant Science & Technology, Huazhong Agricultural University, Wuhan, 430070 China; 2 Department of Agronomy, Faculty of Agricultural Science’s and Technology, Bahauddin Zakariya University Multan, Multan, Pakistan

**Keywords:** Salt stress, SMs, Ca^2⁺^ signaling, Ion homeostasis, Rice, QTL

## Abstract

Salt stress impairs plant growth by disrupting osmotic regulation, ion homeostasis, and oxidative stress management. Plants respond by activating defense mechanisms, including the biosynthesis of secondary metabolites (SMs) such as alkaloids, flavonoids, terpenoids, and glucosinolates (GSLs). Calcium (Ca^2^⁺) signaling is central to these responses, acting as an early stress signal. Ca^2⁺^ influx triggers calcium-dependent protein kinases (CDPKs) and other signaling molecules, which activate stress-responsive genes. SMs are pivotal in mitigating salt stress by promoting osmotic adjustment, maintaining cellular turgor, and modulating ion transporters to reduce Na⁺ uptake and enhance K⁺ retention. This ion homeostasis is closely regulated by Ca^2⁺^ signaling, which influences transport proteins like Na⁺/K⁺ transporters and vacuolar calcium exchangers (e.g., OsCAX1). The crosstalk between SMs and Ca^2⁺^ exhibited a critically important role in salt tolerance, as Ca^2⁺^ influx is an essential trigger for calcium-dependent signaling pathways. Additionally, Ca^2⁺^ signaling regulates the biosynthesis of SMs through transcription factors like MYB and WRKY. These SMs help detoxify reactive oxygen species (ROS) by regulating antioxidant enzymes such as superoxide dismutase (SOD) and catalase (CAT), aided by MAPK signaling cascades. SMs also interact with abscisic acid (ABA) signaling to regulate stomatal closure and stress-related gene expression, enhancing the plant’s resistance to salt stress. Recent meta-QTL analysis has identified key loci involved in SM biosynthesis and Ca^2⁺^ signaling pathways under saline conditions, providing promising targets for breeding salt-tolerant crops. This review explores the molecular mechanisms and regulatory networks of SMs and Ca^2⁺^ signaling in plant salt stress responses, with potential applications in sustainable agriculture.

**Graphical Abstract:**

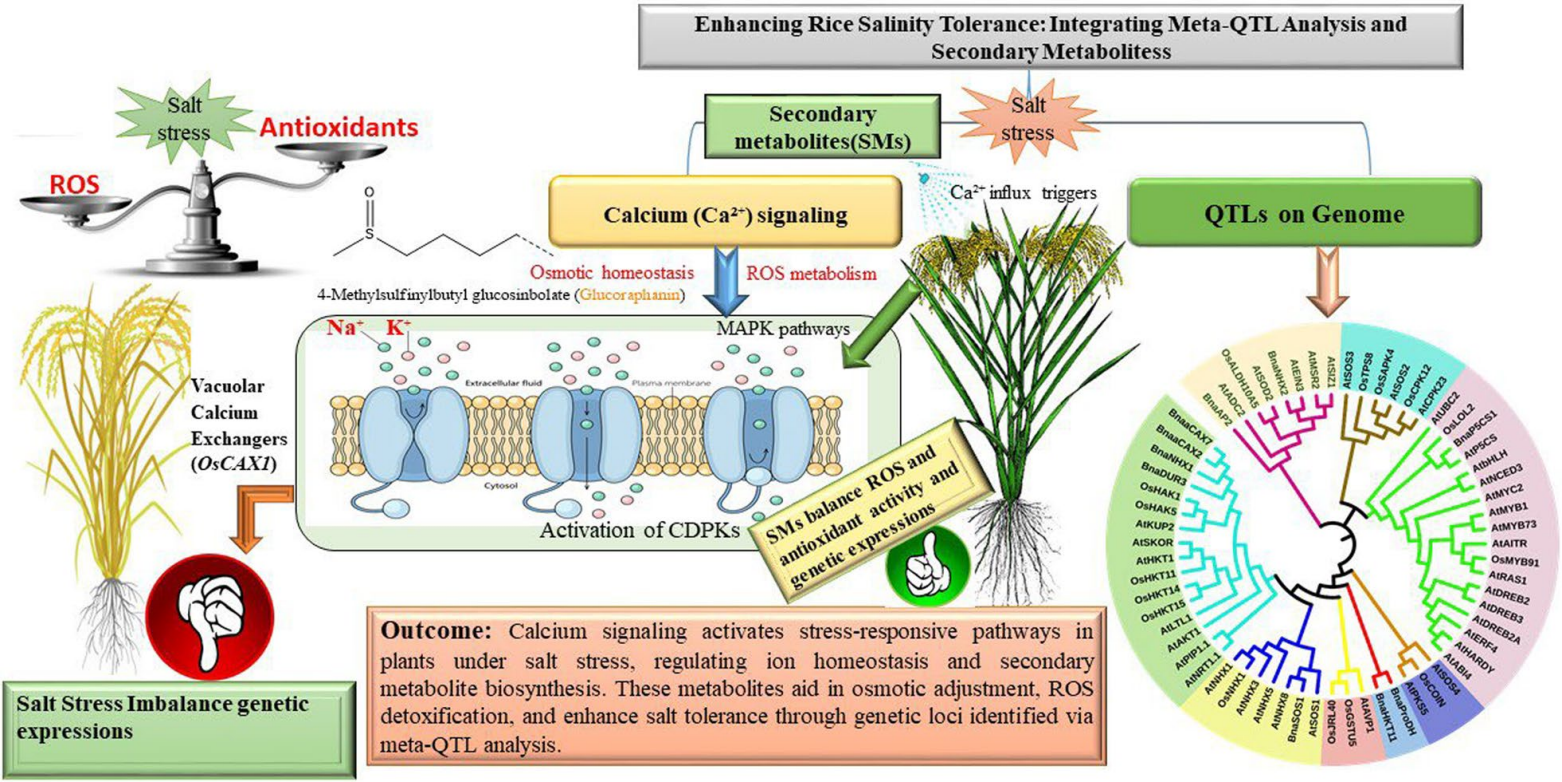

## Introduction

Among the rice’s adaptive mechanisms to mitigate salt stress, secondary metabolites, and calcium signaling pathways have emerged as crucial regulatory components. Secondary metabolites, including flavonoids, alkaloids, and glucosinolates, function as antioxidants, Osmo protectants, and signaling molecules that modulate stress responses (Shiade et al. 2024). These metabolites scavenge reactive oxygen species (ROS), stabilize cellular structures, and regulate ion transport, enhancing salt tolerance (Basit et al. 2022). Concurrently, calcium signaling serves as a key mediator in stress perception and response, facilitating signal transduction through calcium-binding proteins such as calmodulin (CaM), calcium-dependent protein kinases (CDPKs), and calcineurin B-like proteins (CBLs) (Rafiq et al. 2021). These proteins regulate downstream targets involved in ion homeostasis, antioxidant defense, and gene expression, thus reinforcing the plant’s resilience under saline conditions. Recent advances in molecular biology and omics technologies have provided more profound insights into the interplay between secondary metabolites and calcium signaling in salt stress adaptation. Transcriptomic and metabolomic studies have identified key regulatory networks integrating metabolic adjustments with calcium-mediated signal cascades (Ravi et al. 2023). Furthermore, breeding strategies and biotechnological approaches, including CRISPR-based genome editing and marker-assisted selection, are now being explored to manipulate these pathways for improved salt tolerance in rice (Jiang et al. 2020).

Global food security is under increasing pressure due to the need to simultaneously enhance crop productivity, ensure high crop quality, and reduce the negative impacts of agricultural practices on the environment (Basit et al. 2022). The excessive use of chemical inputs, such as fertilizers and pesticides, has been a cornerstone of modern agriculture, with an estimated annual application of 64–65 thousand tons of pesticides and over 2.16 million tons of mineral fertilizers globally (Rafiq et al. 2021). While these inputs have contributed significantly to crop yield improvements, they have also led to adverse environmental and health impacts, including soil degradation, water pollution, loss of biodiversity, and toxicity in food chains (Ramachandran et al. 2021). There is growing interest in developing sustainable agricultural practices to meet the challenge of maintaining high crop productivity while minimizing environmental degradation. These practices include biostimulants and bioprotectants, natural compounds, or microbial preparations that promote plant growth and enhance resilience to biotic and abiotic stresses (Ramireddy et al. 2018). By reducing dependence on synthetic fertilizers and pesticides, biostimulants and bioprotectants offer a more sustainable alternative for managing crop production systems, particularly under stress conditions such as salinity.

Soil salinity is a major abiotic stress severely limiting rice (*Oryza sativa*) productivity, affecting nearly 20% of irrigated land worldwide (Raza et al. 2021). Salinity disrupts ionic balance, induces oxidative stress, and hampers plant growth by interfering with essential physiological processes, such as water uptake and nutrient assimilation. Developing salt-tolerant rice cultivars is a critical strategy for ensuring global food security, particularly in regions where soil salinization intensifies due to climate change and unsustainable irrigation practices (Ahanger et al. 2020).

Secondary metabolites are low molecular weight organic compounds that are not essential for the fundamental plant processes, growth, and development but are necessary for plant protection and adaptation to stress (Verma et al. 2020). These metabolites can be grouped under several categories: alkaloids, phenylpropanoids, terpenoids, and glucosinolates (GSLs). The importance of their function in abiotic stress tolerance, especially under salt stress, is well-developed due to their function in osmotic adjustment, ion homeostasis, and ROS scavenging (Ronzan et al. 2019). Under salt stress, plants accumulate SMs that help to regulate cellular osmotic balance by promoting the synthesis of compatible solutes like proline, glycine betaine, and soluble sugars, which prevent cellular dehydration (Usman et al. 2020). In addition, SMs like GSLs have been shown to modulate the activity of ion transporters, helping to maintain ion homeostasis by limiting the influx of toxic sodium ions (Na^+^) and promoting the retention of beneficial potassium ions (K^+^) (Anjitha et al. 2021) This mechanism prevents ion toxicity and maintains proper cellular functions under saline conditions. Another critical function of SMs in salt-stressed plants is their role in detoxifying ROS, which is overproduced under stress and can cause oxidative damage to cellular structures such as membranes, proteins, and nucleic acids (Jan et al. 2023). SMs enhance the activity of antioxidant enzymes such as SOD, CAT, and POD, which neutralize ROS and protect plants from oxidative stress (Ahmad et al. 2021). Moreover, SMs activate stress signaling pathways, including the mitogen-activated protein kinase (MAPK) cascade, which helps regulate the plant's overall stress response and adaptive capacity (Umesh and Pal 2018).

Among the various SMs, GSLs are particularly noteworthy for their protective role in plants exposed to abiotic stress. These sulfur-containing compounds, primarily found in Brassicaceae species, are known for their ability to influence stress signaling and detoxification processes (Wu et al. 2019). GSLs and their hydrolysis products have been shown to confer enhanced tolerance to salt stress by modulating antioxidant defenses and improving ion homeostasis (Xu et al. 2018). Previous studies suggest that manipulating sulfur metabolism and introducing GSL-related defense strategies from Brassicaceae species could be promising approaches for improving salt tolerance in rice (Khan et al. 2023; Dokaduang et al. 2025).

Recent molecular studies have provided insights into the regulatory networks controlling SM biosynthesis under stress conditions. Transcription factors such as MYB, WRKY, and bHLH have been identified as key regulators of SM pathways, with their expression being modulated by environmental cues such as salinity (Abegaz and Kinfe 2019). Meta-QTL (Quantitative Trait Loci) analysis has further revealed the genetic basis of SM accumulation in response to salt stress, offering new opportunities for breeding salt-tolerant rice varieties by targeting these loci (Kumar et al. 2022a). Despite the known benefits of secondary metabolites (SMs) in improving plant stress tolerance, much of the existing research has focused on their roles in mitigating biotic stresses, with a limited exploration into their specific mechanistic contributions to abiotic stress tolerance, particularly salinity. In rice, a crop susceptible to salinity stress, the exact pathways through which SMs modulate physiological and molecular responses remain underexplored (Bahieldin et al. 2016). While studies have shown that biostimulants can enhance crop resilience, integrating SM functionality with advanced molecular tools such as meta-QTL analysis for developing salinity-tolerant rice varieties is a significant research gap (Böttger et al. 2018). Given the growing impact of salinity on global rice production, there is an urgent need to investigate the protective mechanisms conferred by SMs in rice comprehensively.

This review aims to provide a comprehensive overview of the molecular mechanisms underlying the role of secondary metabolites and calcium signaling in salt stress resilience. By integrating recent findings, we highlight potential breeding targets and genetic interventions that can be leveraged to develop salt-tolerant rice cultivars. Understanding these mechanisms will contribute to stress physiological research and pave the way for sustainable rice production in salt-affected regions.

## Salinity-Induced Physiological Disruptions and Biochemical Countermeasures

When exposed to elevated salinity levels, rice plants undergo a range of morphological, physiological, and biochemical changes, which can lead to plant mortality in severe cases. Salinity impedes vital metabolic functions and adversely affects critical morpho-physiological and yield-related traits such as photosynthesis, plant height, root length, tiller number, panicle length, spikelet count per panicle, grain filling, and overall plant biomass (Isobe and Miyagawa 2022). As a result, there is a substantial reduction in yield. In salt-sensitive species, salinity stress causes contraction and damage to the pericycle. Early-stage seedling exposure to salt stress increases leaf mortality rates (Bundó et al. 2022). Furthermore, panicle sterility significantly diminishes the productivity of rice under salt-stress conditions.

Salinity persuades two primary types of stress in plants: osmotic and ionic. Osmotic stress occurs when root-surrounding salt concentrations exceed the plant's threshold tolerance, while ionic stress arises from excessive Na^+^ influx, leading to higher salt levels in older leaves. This increases Na^+^ concentrations in the vacuole and the cytoplasm, disrupting metabolic activities and leading to cellular death (Chen et al. 2023). Initially, plant growth is curtailed by osmotic stress due to soil salinity, which is later compounded by ionic stress. High soil salinity reduces plant water uptake and triggers various cellular metabolic responses (Jing et al. 2016). During this stage, multiple processes are negatively affected, including cellular expansion, synthesis of cell wall proteins, overall photosynthesis, absorption of photosynthetically active radiation, stomatal conductance, relative water content, transpiration rate, and breakdown of pigments.

**Fig. 1:**
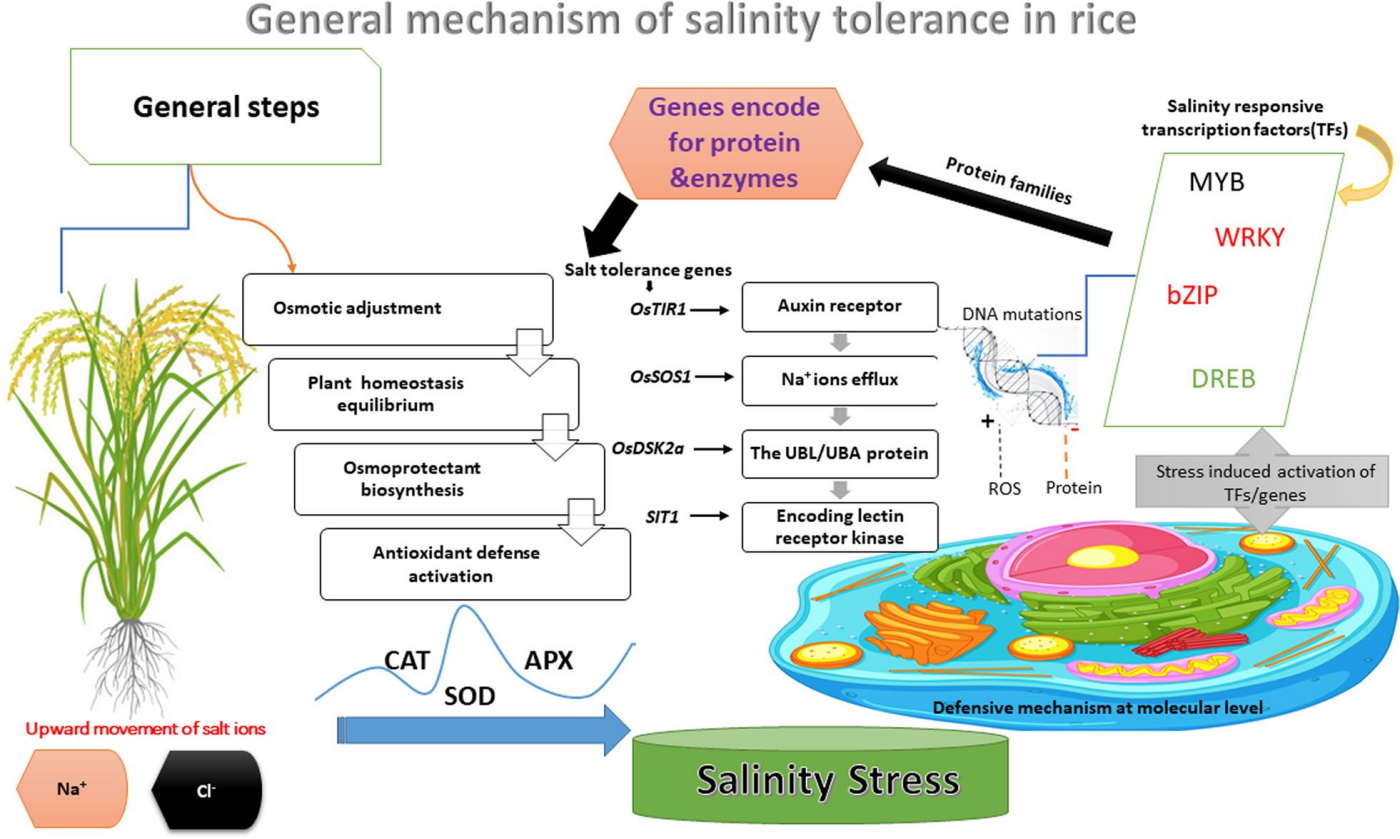
Schematic diagram of salt-tolerant genes and the responsive TFs in enzyme and protein-encoding. Besides, the antioxidant and osmoprotectants role is to mitigate salinity effects in rice

Conversely, the synthesis of compatible solutes and abscisic acid (ABA) increases. Studies have shown that salt stress substantially reduces the levels of carotenoids and chlorophyll in rice leaves (Lakra et al. 2015; Gao et al. 2019). At advanced stages, ion accumulation (especially Na^+^ and Cl^−^) changes the relationship between cotransporters with their ligands: increased ratios of intracellular Na^+^/K^+^ and probably also, to a lesser extent, decreased ratios for Na^+^ coupled Ca^2+^ transport result in enhanced ROS production. It induces abnormal, non-intrinsic ROS production, which increases oxidative stress within cells and leads to a disequilibrium between the generation of ROS and their detoxification (Fig. 1) (Mirdar Mansuri et al. 2020). Rice employs defense mechanisms against salt stress like those observed in other plant species. These mechanisms include the synthesis of antioxidants to neutralize reactive oxygen species (ROS), regulation of ion homeostasis and compartmentalization to mitigate ionic toxicity, accumulation of osmolytes to maintain cellular osmotic balance, and activation of programmed cell death as an adaptive response to severe stress (Kazan 2015).

Plants have evolved a highly sophisticated antioxidant defense system that protects cellular structures from oxidative damage to remove or neutralize ROS (Ahammed et al. 2020). Ji et al. (2024) reported that the rice genotype IR64 exhibited higher levels of AsA and GSH under saline conditions and increased CAT activity, suggesting that these antioxidant molecules played a pivotal role in mitigating salt-induced oxidative stress. The basal portion of the leaf can effectively remove H_2_O_2_ by promoting CAT activity and maintaining APX and GPX at high levels, particularly under saline conditions. Conversely, SOD activity in the apical portion increased to remove O_2_, whereas activities of APX and CAT involved in the removal of H_2_O_2_ decreased under high-temperate conditions. Therefore, both enzymatic and non-enzymatic ROS scavenging mechanisms should work collaboratively to protect the rice against salt-induced oxidative stress. Transportation of salt tolerance in rice roots and its mechanism is controlled by a transcriptional cascade regulated under ROS-dependent SERF1 (Pareek et al. 2020). Recent studies have identified chloride channels (CLCs) as key players in regulating Cl⁻ homeostasis under salinity stress. These channels are involved in the translocation of Cl⁻ from the Golgi to the plasma membrane, facilitating the removal of excess chloride ions from plant cells (Rajappa et al. 2024).

According to the study by Qin et al. (2020) the abiotic stress tolerance in both *indica* and *japonica* rice plants was highly correlated with leaf Na^+^ concentrations, particularly for salinity stress tolerance. A low cytosolic Na^+^/K^+^ ratio is critical for ionic homeostasis, improving photosynthesis and total plant growth (Hasanuzzaman 2022). Under salinity stress, salt-tolerant rice varieties accumulate less Na^+^ in the leaves and shoots than salt-sensitive ones (Kumar et al. 2022b; Liu et al. 2022a). Furthermore, the salt-tolerant rice variety Pokkali effectively minimizes cytosolic Na^+^ accumulation by transiently allowing Na^+^ influx into the cytoplasm before actively sequestering it into vacuoles. In contrast, the salt-sensitive variety BRRI Dhan29 lacks this capability, impairing Na^+^ regulation under salt stress (Tang et al. 2019). Therefore, osmoprotectants and efficient ion homeostasis mechanisms are essential in enhancing rice's tolerance to salinity. Proline, for example, is a compatible solute that protects cells from oxidative stress and maintains cellular integrity under adverse conditions. Similarly, sugars such as trehalose and sucrose play a vital role in sustaining osmotic balance, scavenging reactive oxygen species (ROS), and ensuring cellular function during osmotic stress (Paiva et al. 2019; Liu et al. 2020).

The disaccharide trehalose is also very special for its property of protecting biological structures from dehydration stress. According to (Zhang et al. 2020), the ability of trehalose to be introduced and accumulated in the rice has been enhanced through genetic modification to afford it a certain degree of resistance to the effects of high salt and stretched water. The quaternary ammonium-containing solute glycine betaine has been detected in numerous species. Although rice plants cannot synthesize glycine betaine, the plant can take up glycine betaine externally and accumulate it in the leaves, thus being able to maintain the quantum yield of PS II under salt stress as indicated by (Jing et al. 2016; Rao et al. 2021). If all these mechanisms that help a plant defend itself from salt stress fail, it will succumb to PCD as the last hope of survival (Shabala et al. 2020). It has been demonstrated in other studies that rice roots continuously dying under salt stress are not random but follows a precise regulatory program. This may hinder salt exclusion by preventing further Na^+^ ions from entering inner roots and shoot regions. Another hypothesis is that the plant releases the cells to avoid uncontrollable cell death and associated toxin emissions, which could hurt cells around the globe and nourish beneficial cells (Lei et al. 2017).

### Transcription Factors and Salt Stress Susceptibility

Multiple proteins activate the tolerance mechanisms against salt stress, each performing distinct roles in MDA accumulation, antioxidant and osmoprotectant production, ROS and Na^+^ regulation, and electrolyte leakage. Specific WRKY transcription factors (TFs) suppress the expression of *DREB1B* and *OsNAC1*, thereby increasing susceptibility to salt stress (Islam et al. 2019). Transcription factors can exert both beneficial and detrimental impacts on salt tolerance. Notably, OsCOIN, OsbZIP71, OsbZIP23, OsDREB2A, and OsMYB2 are salt-responsive transcription factors that induce various adaptive changes in rice, comprising the accumulation of osmoprotectants and antioxidants, and enhanced activity of Na^+^ and K^+^ transporters (Mirdar Mansuri et al. 2020). Overexpression of these transcription factors significantly improves seedling survival rates, reduces oxidative damage, and bolsters osmotic regulation (Hossain et al. 2015; Mirdar Mansuri et al. 2020). Conversely, OsWRKY13, a transcription factor with a negative regulatory role, obstructs the expression of salt-responsive genes *SNAC1* and *ERD1*, impeding rice plant growth and development (Tiwari et al. 2016; Zhang et al. 2016). The transcriptional repressor OsWRKY45-2 diminishes the expression of *SNAC1, NCED4, Rab16D*, and *DREB1B* genes, leading to a marked reduction in the survivability of rice cultivars under salt stress (Shabala et al. 2020; Chattopadhyay et al. 2021). Two novel genes (*LOC Os02g49700, LOC Os03g28300*) and five previously identified genes (*OsMYB6, OsGAMYB, OsHKT1;4, OsCTR3, and OsSUT1*) have been linked to grain production and related traits in rice cultivars under saline conditions (McCouch et al. 2016).

A strategy commonly used to increase salt tolerance in rice is illustrated by the fact that sodium ions are primarily sequestered in shoots (Kumar et al. 2015). This is accomplished by sodium exclusion, sequestration of toxic salts in every mature organ, including roots and older leaves, so that leaf senescence will remove these leaves, avoiding direct shedding to waterways, compartmentalization of Na^+^ into vacuoles which makes a communication route for symplastic unloading from the xylem-to-phloem area), and cellular extrusion. Expression of *OsHKT1;1*, *OsHAK10,* and *OsHAK16* expression were elevated in mature rice plant leaves under salinity stress conditions, resulting in increased shoot Na^+^ ions transport (Campbell et al. 2017).

Conversely, a decrease in the activity of *OsHKT1;5* and *OsSOS1*, responsible for Na^+^ removal from xylem vessels in roots, results in higher Na^+^ concentration in older leaves than younger ones. HKT transporters in rice remove excess Na^+^ from the xylem, safeguarding photosynthesis-dependent leaf tissues from salt-induced damage (Table 1). During salt stress, the K^+^ transporter genes *OsHAK1* and *OsHAK5* are activated to enhance K^+^ absorption and transfer, maintaining a favorable K^+^/Na^+^ ratio (Patishtan et al. 2018). When cytosolic Na^+^ levels rise, Na^+^/H^+^ antiporters transport Na^+^ into vacuoles to prevent enzyme-damaging concentrations. This antiporter activity is stimulated by increased salt levels (Cui et al. 2018). Vacuolar H^+^-ATPase and vacuolar H^+^-translocating pyrophosphatase regulate Na^+^/H^+^ exchange in the vacuole. Variation in the expression level of vacuolar transporters might mean a tremendous opportunity to enhance salt tolerance efficiency in rice (De Leon et al. 2017).

**Table 1:** Mechanisms of hormonal and secondary metabolite Interactions in salinity stress amelioration in rice

Plant Hormone	NaCl Concentration (mmol/L)	Mechanism of action (Morphological changes)	Impact on biochemical changes	Molecular/Genetic mechanisms	Secondary metabolites	Interaction between hormones & metabolites	References
Auxin (IAA)	0, 50, 100, 150, 200	Induces root elongation, enhances lateral root formation, promotes leaf area expansion, and increases panicle development	Increases chlorophyll content, reduces ROS accumulation, stabilizes membrane integrity, and enhances osmotic regulation via soluble sugars	Upregulates auxin signaling genes (TIR1, AFB2) and enhances stress signaling pathways to regulate root architecture and cell elongation under salt stress. Improves ion homeostasis	Flavonoids, phenolics	Flavonoid biosynthesis pathways activated by auxin modulate ROS scavenging and stress tolerance. Auxin-induced flavonoid accumulation enhances antioxidant defenses, reducing Na^+^ toxicity	Ahmad et al. (2018)
Gibberellic acid (GA)	0, 50, 100, 150, 200	Promotes seedling growth, increases panicle length, enhances tillering, and promotes grain filling under salt stress	Enhances proline accumulation, increases potassium, calcium, and magnesium uptake, improves osmotic balance. Elevates soluble sugar content, especially under high salinity	Upregulates GA biosynthesis genes (GA3ox, GA20ox), reduces GA catabolism (GA2ox), enhancing growth under salt stress. Modulates stress-responsive gene expression (e.g., *DREB*, *LEA*)	Triterpenoids, saponins	GA enhances saponin synthesis which aids in reducing cellular damage. Triterpenoids reduce oxidative damage by interacting with GA-regulated stress genes, promoting better growth under salinity	Arbona et al. (2013)
Abscisic acid (ABA)	0, 50, 100, 150, 200	Reduces stomatal opening to conserve water, increases root biomass, and enhances water use efficiency under salt stress	Enhances the accumulation of proline, soluble sugars, and compatible solutes. Increases antioxidant enzyme activity (SOD, CAT) to reduce oxidative damage	Upregulates ABA biosynthesis genes (*OsNCED*, *OsABA1*), activates stress-responsive genes (*RD29A*, *DREB2*) to mitigate water loss and ionic imbalance under salt stress	Alkaloids (e.g., nicotine)	Alkaloid accumulation induced by ABA helps improve salt tolerance by stabilizing cell membranes and reducing Na^+^ toxicity. Alkaloids interact with ABA-mediated osmotic regulation, enhancing overall plant resilience	Jiang et al. (2020); Muhammad Aslam et al. (2022)
Salicylic acid (SA)	0, 50, 100, 150, 200	Promotes seed germination, increases root and shoot elongation, boosts panicle number, and enhances yield	Increases carbohydrate and protein content, reduces Na + accumulation, and improves potassium and calcium uptake. Enhances antioxidant enzyme activities (SOD, POD, CAT)	Upregulates SA biosynthesis genes (*ICS1*, *PAL*), activates defense genes (*PR1*, *WRKY*), and enhances stress-related pathways. Promotes salinity tolerance by improving ionic balance and reducing ROS	Glucosinolates, terpenoids	Glucosinolates interact with SA to enhance stress tolerance through ROS scavenging. SA-induced glucosinolate accumulation reduces oxidative stress and regulates ion homeostasis, improving overall stress tolerance	Abdi et al. (2022)
Brassinosteroids (BR)	0, 50, 100, 150, 200	Increases root and shoot elongation, improves biomass production, and promotes panicle development under salt stress	Enhances proline accumulation, increases chlorophyll content, reduces lipid peroxidation, and improves antioxidant system (SOD, APX, CAT)	Upregulates BR signaling genes (*BZR1*, *BES1*), enhances antioxidant defenses, reduces ROS accumulation, and promotes ion transporters (HKT1) to maintain ion balance under salinity	Phenolic compounds, lignans	Brassinosteroids increase the production of phenolic compounds, which act as antioxidants. This interaction mitigates oxidative stress and enhances salt tolerance. Phenolics stabilize cellular structures under salinity stress, aiding in better growth	Manghwar et al. (2022)
Cytokinins (CK)	0, 50, 100, 150, 200	Increases shoot elongation, enhances leaf expansion, and promotes tiller formation under salt stress	Improves nitrogen assimilation, increases protein synthesis, reduces chlorophyll degradation, and maintains cell division	Upregulates cytokinin biosynthesis genes (*IPT*, *LOG*), increases cell division, modulates cytokinin-responsive genes (*ARR1*, *AHK4*), and promotes salinity tolerance through improved growth	Tannins, flavonoids	Cytokinins enhance flavonoid and tannin production to protect cells from oxidative damage. These metabolites, in turn, help cytokinin-regulated growth under salt stress by reducing ROS and improving cell wall integrity	Ahmad et al. (2018)
Ethylene (ET)	0, 50, 100, 150, 200	Enhances root growth, increases lateral root formation, and promotes plant adaptation to saline environments	Reduces lipid peroxidation, enhances antioxidant activity (SOD, CAT, APX), and improves potassium uptake	Upregulates ethylene-responsive genes (*ERFs*, *EIN3*), activates stress-responsive pathways, and enhances root system development to improve salt tolerance under saline conditions	Flavonoids, alkaloids	Ethylene promotes flavonoid and alkaloid synthesis, which helps to stabilize cellular structures and reduce salt-induced damage. These metabolites enhance ET-regulated stress responses, improving tolerance to salinity	Arbona et al. (2013)
Jasmonic acid (JA)	0, 50, 100, 150, 200	Promotes root lengthening, root branching, and seedling vigor under salt stress conditions	Increases accumulation of soluble sugars, proline, and secondary metabolites (e.g., terpenoids, phenolic acids), and improves antioxidant status	Upregulates JA biosynthesis genes (*LOX*, *AOC*), activates defense-related genes (PDF1.2, PR proteins), reduces ROS accumulation, and enhances growth under salt stress by modulating gene expression in the defense network	Terpenoids, phenolic acids	JA enhances terpenoid and phenolic acid synthesis, which contributes to the enhancement of salt tolerance. These metabolites help in reducing oxidative damage and improving overall plant defense mechanisms under saline conditions	Jiang et al. (2020)
Melatonin (MT)	0, 50, 100, 150, 200	Enhances root growth, leaf chlorophyll content, and overall plant size, promoting salt stress recovery	Increases antioxidant enzymes (CAT, SOD), reduces lipid peroxidation, increases soluble sugars, and modulates osmotic pressure	Upregulates melatonin biosynthesis genes (T5H, SNAT), regulates ROS through antioxidant mechanisms, and improves photosynthetic efficiency, promoting better growth and stress recovery under saline conditions	Indole alkaloids, flavonoids	Melatonin interacts with flavonoids and indole alkaloids, promoting salt tolerance through enhanced antioxidant activity and ROS scavenging. These metabolites mitigate oxidative damage and help plants recover under salinity stress	Zhang et al. (2025)

### Disruption of Ion Homeostasis in Rice

Soil salinity involves the accumulation of various salts, including sodium chloride, magnesium sulfates, and bicarbonates of magnesium and calcium (Suzuki et al. 2016). Rice, susceptible to salinity during its early growth phases, experiences reduced yield efficiency under saline conditions. The vulnerability of rice to salt stress is pronounced during its initial growth and reproductive stages. Salt-laden water transports harmful ions into the plant's vascular root system via two primary pathways: apoplastic and symplastic. The apoplastic pathway facilitates the excessive accumulation of Na^+^ in the shoots, notably in mature leaves, under saline conditions. The high-affinity potassium transporter (HKT), a Na^+^/K^+^ symporter, plays a crucial role in mediating the transfer of Na^+^ and K^+^ across plant cell membranes (Kumar et al. 2022a). Excessive sodium disrupts potassium uptake at the root surface, as Na^+^ ions interfere with K^+^ absorption due to their chemical similarity. Under salt stress, an influx of Na^+^ ions into the plant exacerbates the internal concentration of Na^+^, adversely affecting the plant's physiological processes (Wang et al. 2015). This is primarily because Na^+^ competes with K^+^ for enzyme activation and protein synthesis, undermining plant health and productivity.

High Na^+^ and Cl^−^ levels commonly induce stress in crop plants due to soil salinity, as summarized in (Fig. 1). Recent research emphasizes the role of sodium transporters such as the NHX (Na⁺/H⁺ exchangers) and HKT (high-affinity potassium transporters) in limiting Na⁺ uptake at the roots and sequestering Na⁺ into vacuoles, thereby reducing its toxic effects on cellular processes (Chen et al. 2023). Ion transporters play a pivotal role in salt tolerance by mediating the transport of detrimental ions at plants' organ and cellular levels. A key focus of salinity tolerance research involves identifying transporters that limit Na^+^ influx into cells. Na^+^ and K^+^ often utilize the same transport pathways, resulting in a competitive interaction that can hinder the uptake of potassium ions, which are vital for activating key catalytic enzymes (Ma et al. 2016).

Variations in plant glucosinolate levels and distribution are closely linked to their developmental stages (Seo et al. 2017). Under salt stress, glucosinolate accumulation is often more pronounced in florets than in young, fully expanded leaves, possibly due to enhanced de novo synthesis or increased translocation via the phloem (Raees et al. 2023). The reduced glucosinolate production in older leaves, followed by their redistribution to other plant parts, may explain the observed variations in glucosinolate concentrations under salinity stress. Studies on the halophyte *Thellungiella* have shown that glucosinolate profiles shift across developmental stages and plant organs upon salt treatment, indicating selective de novo synthesis and spatial redistribution of glucosinolates (Shawon et al. 2020). The glucosinolate-myrosinase system plays a vital role in stress adaptation. Myrosinases, stored separately from glucosinolates, hydrolyze them upon cellular damage, leading to the release of breakdown products such as isothiocyanates and nitriles (Sun et al. 2023).

These metabolites contribute to ion homeostasis by modulating ion transporter activity and mitigating oxidative stress-induced ion imbalances. Salt stress causes membrane lipid peroxidation, leading to increased myrosinase release and subsequent glucosinolate degradation (Tang et al. 2023). The degradation of glucosinolates (GSLs) during salt stress has been linked to membrane loss, which results from oxidative stress-induced lipid peroxidation that compromises membrane integrity. This disruption allows for the release of myrosinase, an enzyme responsible for GSL breakdown (Khan et al. 2023). This breakdown has been linked to Na⁺/K⁺ homeostasis, as glucosinolate-derived metabolites can regulate the expression of ion transporters like SOS1 (Salt Overly Sensitive 1), NHX1 (Na⁺/H⁺ exchanger), and HKT1 (High-Affinity K⁺ Transporter), which are critical for maintaining intracellular ion balance.

Salt stress also induces metabolic shifts, leading to an increased accumulation of secondary metabolites such as flavonoids and phenolics, which may compete with glucosinolates for metabolic resources and influence ion transport processes (Jiang et al. 2020). This metabolic reprogramming is crucial for salt tolerance, as glucosinolate-derived metabolites not only contribute to oxidative stress alleviation but also indirectly support Na⁺ compartmentalization and K⁺ retention. Additionally, salt-induced fluctuations in glucosinolate concentrations correlate with disruptions in plant growth and reproductive processes. Reduced seed setting and delayed flowering in *Brassica napus* under salt stress are associated with glucosinolate imbalances, which may affect the plant’s ability to regulate ion fluxes during reproductive development (Zhang et al. 2022).

**Fig. 2:**
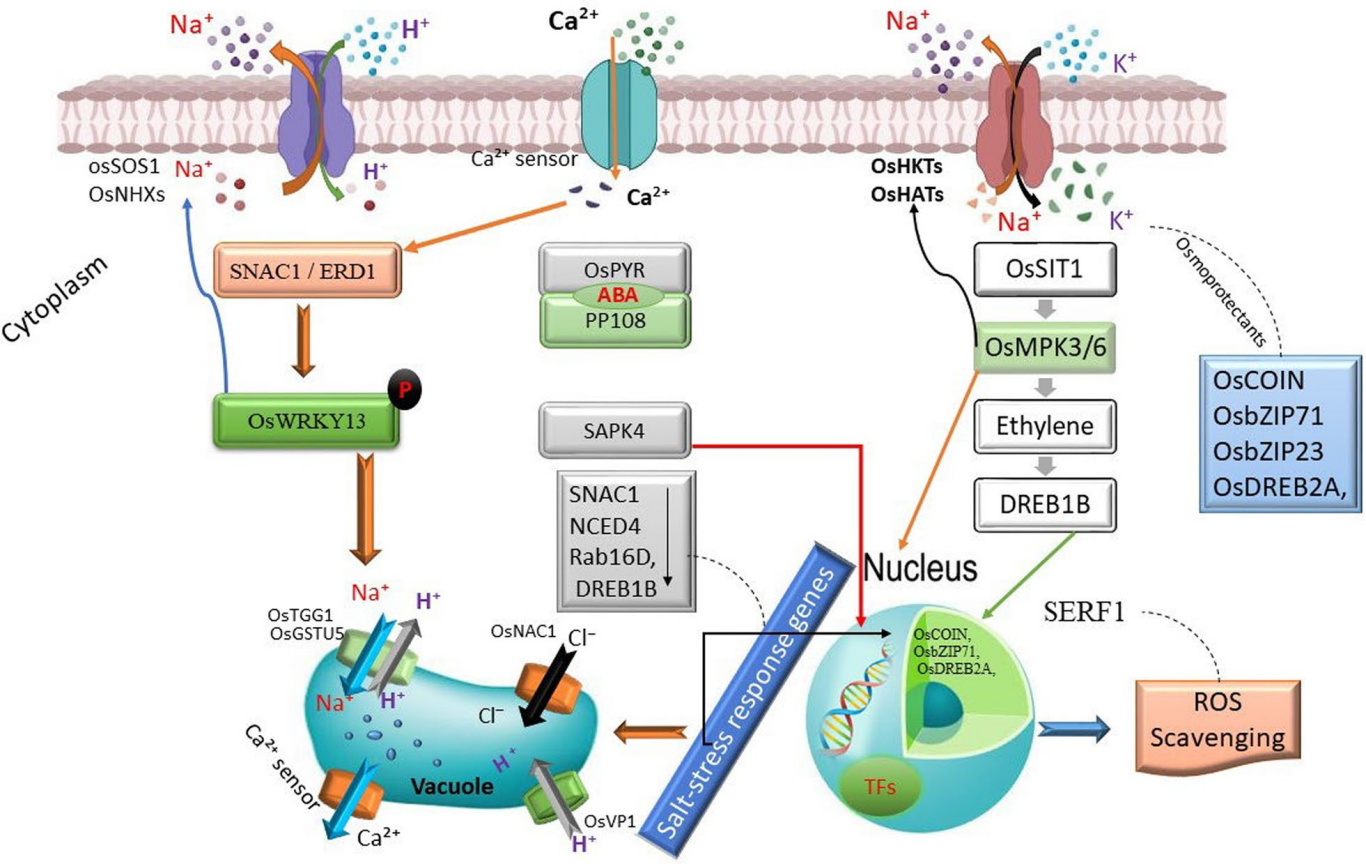
Salt stress tolerance in *Oryza sativa*: a diagram depicting the responses of rice plants to salt stress conditions demonstrates the complex effects of high salinity on plant health, including morphological, physiological, biochemical, and yield-related responses

At the molecular level, salt stress influences glucosinolate biosynthesis through the MAP kinase and calcium signaling pathways, which also regulate ion transport mechanisms. The transcription factors MYB28 and MYB29, which control glucosinolate biosynthesis, are downregulated by ABA signaling under salt stress (Wang et al. 2025). Jogawat et al. (2021); Khan et al. (2021) reported that salt stress can alter the biosynthesis and breakdown of glucosinolates, potentially affecting the balance between defensive and metabolic pathways in plants. Since ABA signaling plays a central role in Na⁺/K⁺ homeostasis by regulating the expression of SOS, NHX, and HKT transporters, this suggests an indirect but significant link between glucosinolate metabolism and ion homeostasis. Moreover, enzymes such as cytochrome P450s and glutathione-S-transferases, which participate in glucosinolate biosynthesis, are regulated by salt stress, further reinforcing the connection between glucosinolate metabolism and salinity adaptation (Najeeb et al. 2020).Overall, glucosinolates contribute to salt stress responses not only as defense compounds but also as modulators of ion homeostasis. Their degradation products influence the activity of key ion transporters, assisting in Na⁺ exclusion and K⁺ retention, while stress-induced shifts in glucosinolate metabolism impact ion transport indirectly through ABA signaling. Further research is needed to dissect the regulatory network linking glucosinolate metabolism to ionic balance and to explore its potential for enhancing salt tolerance in crops. It is also crucial to explore other potential roles of glucosinolates, particularly in improving disease resistance under salt stress, rather than solely focusing on their activation of defense pathways (Fig. 2).

## Role of Plant Secondary Metabolites in Enhancing Salinity Tolerance in Rice Mechanism

**Fig. 3:**
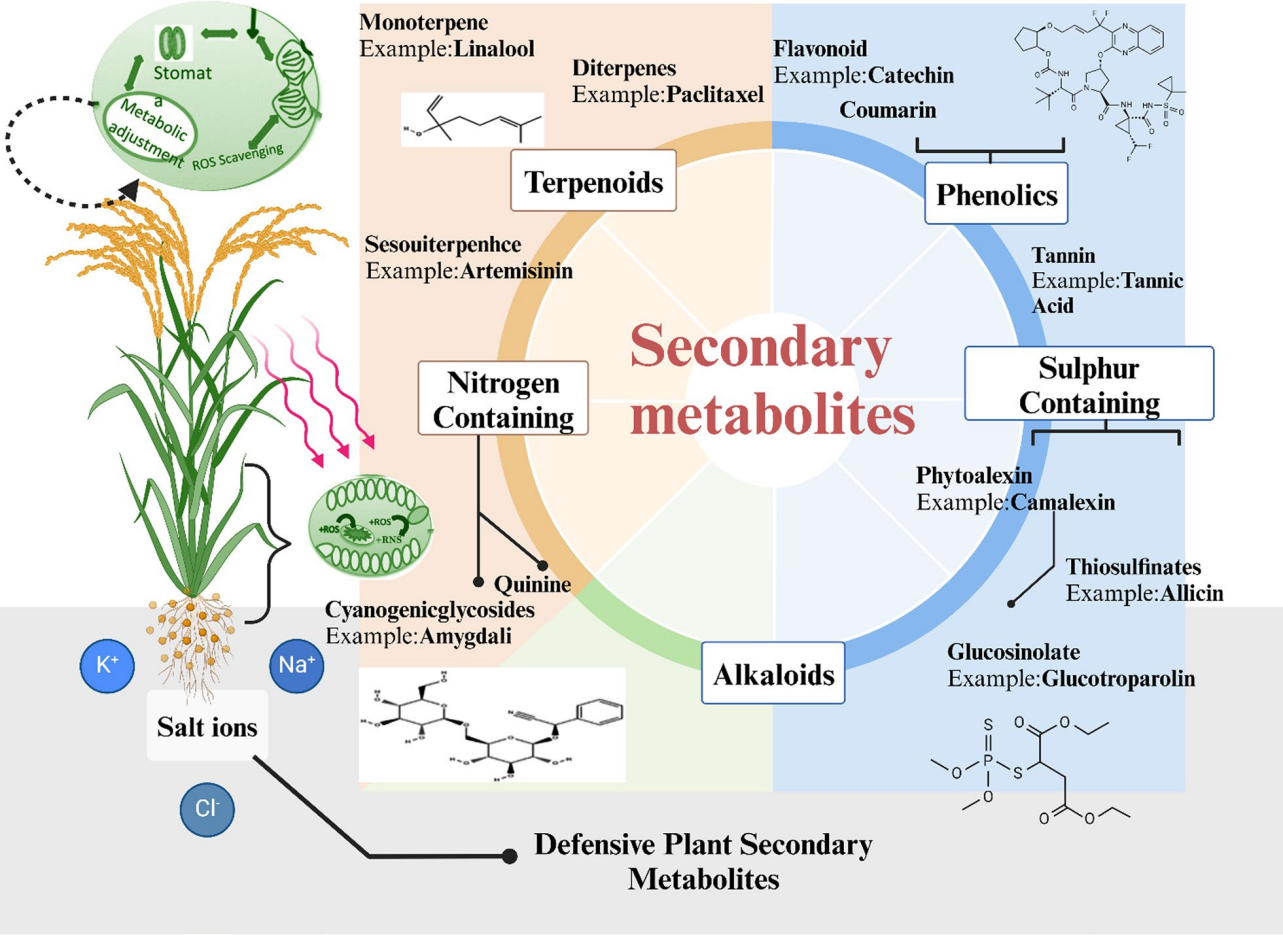
Structural diversity of key defensive secondary metabolites in plants, including alkaloids, phenolics, and terpenoids. Representative compounds such as glucosinolates (sulfur-containing compounds involved in stress response), flavonoids (antioxidant-rich phenolics), and phytoalexins (inducible antimicrobial metabolites) are highlighted. Each structure is depicted with its core functional groups, emphasizing biochemical properties and roles in plant defense against abiotic and biotic stresses. The illustration provides a comparative visualization of these metabolites, showcasing their chemical frameworks and potential interactions with environmental stressors

Plant secondary metabolites (SMs) are organic compounds produced via secondary metabolic pathways, distinct from the primary metabolic processes essential for growth and development. These metabolites are involved in various stress responses, including enhancing salinity tolerance in rice (Acquavia et al. 2021). Their roles are critical in enabling plants to adapt to multiple abiotic stressors, particularly salt stress. The efficacy of these metabolites is closely linked to their evolutionary functions, which include attracting beneficial organisms and providing protection against environmental stressors (Fig. 3). For instance, SMs can enhance a plant's resilience to salt-induced osmotic and ionic stress by modulating physiological processes and activating protective mechanisms (Adnan et al. 2017; Verma et al. 2020). This resilience is attributed to the structural diversity of key defensive secondary metabolites in plants, including alkaloids, phenolics, and terpenoids. Representative compounds such as glucosinolates (sulfur-containing compounds involved in stress response), flavonoids (antioxidant-rich phenolics), and phytoalexins (inducible antimicrobial metabolites) contribute to stress tolerance by interacting with environmental stressors. These metabolites possess distinct biochemical properties, as highlighted by their core functional groups, which play crucial roles in plant defense against both abiotic and biotic challenges. As an abiotic challenge, the activation of diverse secondary metabolites in response to salt stress is facilitated by conserved defense mechanisms in plants. This process involves the action of defense proteins and microbe-associated molecular patterns (MAMPs), which are recognized by pattern recognition receptors (PRRs) (Piasecka et al. 2015; Pillai et al. 2020). Through these mechanisms, plants can enhance the biosynthesis of secondary metabolites that help mitigate the detrimental effects of salinity, thereby improving their resilience. Due to the wide variety of SMs and their roles in plant defense, multiple criteria have been established for their classification, including their biosynthetic pathways and their involvement in stress-related responses. Secondary metabolites, including phenolic compounds, flavonoids, terpenoids, alkaloids, and glycosides, play crucial roles in mitigating the effects of salt stress in rice through several key mechanisms. These metabolites contribute to the plant's resilience by protecting cellular structures, maintaining ion balance, and enhancing stress-related signaling pathways.

One of the primary responses involves phenolic compounds, particularly salicylic acid (SA), which act as potent antioxidants. Salinity stress leads to excessive production of reactive oxygen species (ROS), which can damage cellular structures like membranes and proteins. SA plays a crucial role by scavenging ROS, thus reducing oxidative damage (Celedon et al. 2017). In addition to its antioxidant properties, SA activates key enzymes like superoxide dismutase (SOD) and catalase (CAT), which further neutralize harmful ROS, stabilizing cell membranes and improving ion homeostasis (Chen et al. 2017). This ensures better plant resilience in saline environments by protecting cells from oxidative stress and maintaining membrane integrity. Flavonoids, another group of secondary metabolites, contribute to salinity tolerance by regulating ion homeostasis and maintaining redox balance. Salinity disrupts the sodium (Na⁺) and potassium (K⁺) balance in plant cells, essential for cellular function. Flavonoids enhance the activity of ion transporters such as Na⁺/H⁺ antiporters, which help remove excess sodium from the cytoplasm and maintain an optimal K⁺/Na⁺ ratio (Dubois et al. 2018).

Flavonoids, terpenoids, and alkaloids are crucial secondary metabolites that significantly enhance salinity tolerance in rice. Flavonoids boost antioxidant defenses by regulating non-enzymatic antioxidants like ascorbate and glutathione, thus reducing oxidative damage caused by salinity stress and ensuring the continuity of cellular processes (Kim et al. 2020; Sohag et al. 2020). Terpenoids, particularly those derived from abscisic acid (ABA), stabilize membranes and regulate osmoregulation by controlling stomatal closure, which reduces water loss through transpiration under osmotic stress. They also protect cell membranes by maintaining the integrity of lipid bilayers often disrupted in saline environments, preventing dehydration (Sabagh et al. 2020). Alkaloids are essential in regulating ion transport and modulating stress signaling pathways. They help counteract the toxic effects of excessive sodium ion uptake by influencing ion channels and pumps, such as H⁺-ATPases, which actively transport sodium out of the cytoplasm, restoring ion balance. Additionally, alkaloids modulate hormonal signaling pathways, including those involving ethylene and jasmonic acid (JA), which are key in activating stress defense mechanisms. This dual action of alkaloids in ion regulation and stress signaling significantly enhances rice's ability to cope with saline environments (Najeeb et al. 2020; Usman et al. 2020).

Finally, glycosides play a role in detoxification and stress response activation. Salinity often leads to the accumulation of toxic ions, which can interfere with metabolic processes (Sheteiwy et al. 2019). Glycosides, such as cardenolides and saponins, bind to these toxic ions, reducing their harmful effects. They also activate stress-related gene expression by triggering transcription factors like DREB and NAC, which regulate abiotic stress responses (Mousavi et al. 2020). This detoxification process, combined with the activation of stress defense pathways, helps rice plants mitigate the toxic effects of salinity and improve their overall stress tolerance. In summary, secondary metabolites in rice contribute to salinity tolerance by engaging in various protective mechanisms, including ROS scavenging, ion homeostasis, membrane stabilization, osmoregulation, and stress signaling. These compounds work together to ensure that rice plants maintain cellular integrity and functionality under saline conditions, ultimately enhancing their ability to survive and thrive in stressful environments.

### Glucosinolates (GSLs)

Glucosinolates (GSLs) are sulfur and nitrogen-based secondary metabolites derivative of amino acids, primarily present in Brassica crops such as cabbage, broccoli, and oilseed rape (Ben Ammar et al. 2023). GSL-derived bioactive metabolites are essential for plant defense, human health benefits, and the characteristic flavor profiles of specific Brassica vegetables (Anjitha et al. 2021). miRNAs play a crucial role in regulating the biosynthesis and metabolic pathways of glucosinolates (GSLs), acting as key modulators of gene expression to enhance GSL accumulation, which contributes to plant defense mechanisms under abiotic stress conditions. For instance, miR395 has been reported to regulate sulfate assimilation, influencing GSL biosynthesis in response to sulfur deficiency, while miR824 targets AGAMOUS-LIKE transcription factors, modulating aliphatic GSL accumulation under stress conditions (Ding et al. 2024). These miRNA-mediated regulations highlight their significance in fine-tuning GSL metabolism to enhance stress resilience in plants.

Accordingly, large-scale studies have been conducted to elucidate the broad areas in which glucosinolates and their degradation products function in Brassica plants under diverse physiological states. Previous studies (Eom et al. 2022; Chen et al. 2023) have inspected the effects of GSL composition or profile on plants’ health benefits/resistance under stress. Structurally, GSLs fall into three standard categories, including aliphatic, indolic, and aromatic, with greater than 200 isomers identified for each type characterized by one-of-a-kind substituents (Chung et al. 2019; Chen et al. 2023). Many studies document the critical function GSLs likely play in responses to multiple abiotic stresses, especially salt stress tolerance, as is strongly supported by the literature.

Even though the knowledge on abiotic stress and its effects on GSL metabolism is scarce, it was already revealed that glucosinolates form a class of secondary metabolites consisting of nitrogen- and sulfur-based compounds primarily occurring in Capparales plants as well as Brassicaceae species such as Brassica crops or *Arabidopsis thaliana* (Doghri et al. 2022; Eom et al. 2022). These compounds are stable for chemical transformation until tissue disruption in a plant activates an enzyme called myrosinase. This activation leads to several breakdown products, including isothiocyanates, nitriles and thiocyanates, epithionitriles, and oxazolidines (Shakour et al. 2022). The subsequent breakdown depends on pH, ferrous ions, and interactions with myrosinase-associated proteins. Isothiocyanates have received considerable attention because they defend plants against pathogens and herbivores.

Moreover, humans' dietary intake of glucosinolate-containing vegetables such as broccoli, kale, and Brussels sprouts has been associated with putative anti-cancer properties (Heinze et al. 2018). Nevertheless, the overall physiological significance of glucosinolates and their metabolites in plant biology has not been completely elucidated. The most important thing is the glucosinolate fusion in the myrosinase system, which regulates plant–herbivore and plant-pathogen interactions. In other studies, glucosinolates have been proposed to function as storage compounds of essential nutrients, including nitrogen and sulfur (Lv et al. 2022). Increased exposure to sulfur has been shown to reduce the glucosinolate sink capacity in Brassica species.

#### Impact of Abiotic Stimuli on GSLs

**Fig. 4:**
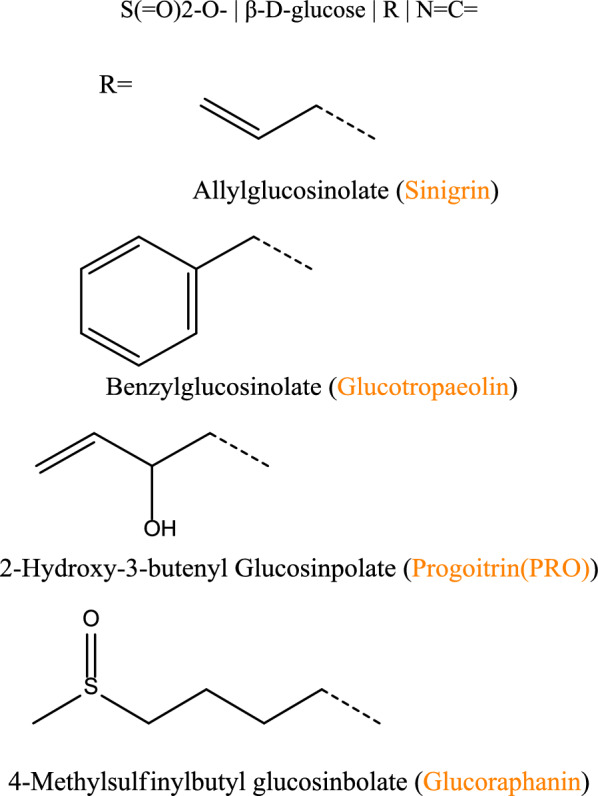
Chemical structures of glucosinolates and their key variants, including benzyl glucosinolate, 2-hydroxy-3-butenyl glucosinolate, and 4-methylsulfinylbutyl glucosinolate. These sulfur-containing secondary metabolites play a crucial role in plant defense against abiotic and biotic stresses. Structural variations arise from different R-groups, influencing their biological activity and ecological functions

The detrimental effects of salt stress on primary metabolic activities, including photosynthesis, growth, and antioxidant metabolism, are extensively documented across various studies (Hasanuzzaman 2022). Within the realm of plant stress, it is typical for secondary metabolism to intensify due to the constraints on growth that exceed those imposed by photosynthesis. These changes come at the expense of converting fixed carbon into primary growth later in ontogeny, taking up available shared resources for synthesizing secondary metabolites. Also, environmental conditions are significantly involved in the modulation of secondary metabolism. Earlier studies have indicated that light, temperature, salt, and drought can alter glucosinolate composition and structure, as shown in the structural diagram below (Gai et al. 2020; Hao et al. 2022).Glucosinolates are sulfur-containing secondary metabolites that play a crucial role in plant defense against environmental stresses. Their structural diversity, governed by variations in the R-group, influences their biochemical properties and defensive potential, the core glucosinolate structure alongside key variants, including benzyl glucosinolate, 2-hydroxy-3-butenyl glucosinolate, and 4-methylsulfinylbutyl glucosinolate, highlighting their functional diversity in plant stress responses (Fig. 4). Nevertheless, the precise physiological mechanism of all these components influencing abiotic stress tolerance in plants is not clearly understood.

In contrast to the above-mentioned, RNA interference of aliphatic glucosinolates in *Arabidopsis* plants alters proteins and metabolites involved in critical physiological processes such as photosynthesis, oxidative stress, and hormone metabolism (Peterson et al. 2018). Both studies indicate a strong connection between physiological states and glucosinolate metabolism under abiotic stress conditions (Satasiya et al. 2024). Understanding biochemical alterations in plants during abiotic stress and the subsequent ecological ramifications on plant–herbivore interactions is crucial. This review discusses how various abiotic factors, specifically salinity, affect glucosinolate biosynthesis.

To alleviate salt stress on plants, one of the strategies is to synthesize secondary metabolites that have functions in defending against constraints from the environment and growth. This approach has undergone extensive research, and the field is currently reaching a new height because of progress in metabolite profiling and its quantification (Poveda et al. 2020). The changes in metabolites associated with plant stress responses have recently been expanded. Since then, the essential roles of these compounds have been shown, such as in salt-stress-tolerance mechanisms (Kim et al. 2020). The formation of chemicals typically involved in plant defense is moderated by diurnal or circadian rhythms, which consider existing environmental growth constraints. These data suggest that the secondary metabolite profiles of numerous components are misleading, which significantly complicates specific mappings of valuable compounds in salt stress response (Zhao et al. 2020).

Plant metabolism is influenced by a myriad of factors, including climatic conditions such as seasonal changes, light intensity, water availability, temperature variations, carbon dioxide levels, air and soil pollution, biotic factors like plant species, genotype, pests, pathogens, and competitive flora, and agronomic factors like soil quality, fertilizer types, pesticide application, cultural practices, and habitat manipulation (Park et al. 2021). It is widely accepted among researchers that salt stress can affect around 200,000 secondary metabolites in plants, which represent over 50% of the estimated total. These chemicals serve various defensive functions in plants, including antioxidants, antifungal, antibacterial, herbivore deterrent, and stress-inducible phytohormone activities (Alotaibi et al. 2023). Plant defense mechanisms are characterized by a diverse array of well-documented metabolites, including terpenoids, alkaloids, phenolics, steroids, flavonoids, tannins, and other potentially unidentified compounds (Fig. 1) (Poveda et al. 2020; Pereira et al. 2023). These plants produce secondary metabolites such as glucosinolates, carotenoids, flavonoids, phenolic acids, and alkaloids in response to abiotic stressors like drought, salinity, cold, and high temperatures. Such metabolites enhance the plant's antioxidant capabilities to counteract oxidative stress, a secondary effect of abiotic stressors. Increased levels of these defense-related secondary metabolites are often noted following exposure to both biotic and abiotic stresses. The extensive biological properties of these phytochemicals and their degradation products, including antioxidant, anticancer, antibacterial, anti-inflammatory, antidiabetic, and neuroprotective activities, have been extensively documented concerning human dietary benefits (Podda et al. 2019).

Consequently, abiotic stressors that alter metabolite production quantity and quality can significantly affect animals' and humans' nutritional value and overall well-being. It is crucial, therefore, to manage the growth conditions of crops meticulously to maximize metabolite accumulation and maintain desirable metabolite profiles in the edible parts of plants. Preserving the inherent characteristics of host plants, such as color, aroma, flavor, and health-promoting properties, is vital. The accumulation of specific glucosinolates (GSLs) in Brassica species has negatively impacted their nutritional quality (Rao et al. 2021). Nonetheless, these specialized compounds synthesized under stress conditions play a role in mitigating adverse growth environments through various mechanisms (Rezayian and Zarinkamar 2023). Previous studies have demonstrated the biological significance of glucosinolates, sterols, terpenes, and flavonoids in the metabolic adaptation of plants to abiotic stresses (Rasheed et al. 2021). Although our understanding of how plants respond to stress is not yet fully comprehensive, recent progress in agricultural techniques and analytical chemistry has made it possible to identify the individual metabolites that are built up in certain physiological situations (Salami et al. 2023).

#### Glucosinolate Composition Changes Under Salt Stress: Plant Adaptation Insights

Salt is an important abiotic factor impacting plant physiological and developmental processes (Fig. 4). It modulates secondary metabolism, generally related to changes in signaling molecule levels, oxidative stress, and intermediary pathways (Rezayian et al. 2018). Glucosinolates are significant secondary metabolites, and a remarkable up-regulation of their biosynthesis occurs when the concentration of salinity stress surpasses tolerance thresholds. Under salt stress, plants activate complex molecular responses to minimize damage, with a key mechanism regulating genes involved in glucosinolate (GSL) biosynthesis. The genes *OsTGG1* (Oryza sativa thioamylase 1) and *OsTGG2* (Oryza sativa thioamylase 2) are responsible for hydrolyzing specific GSLs, particularly those in the aliphatic class, into active derivatives such as isothiocyanates. These derivatives play critical roles in detoxifying reactive oxygen species (ROS), helping to neutralize ROS like hydrogen peroxide (H₂O₂) and superoxide radicals (O_₂_⁻). The increased production of isothiocyanates through the upregulation of *OsTGG1* and *OsTGG2* enhances the antioxidant capacity of the plant, thereby reducing oxidative damage to membranes, proteins, and DNA under high salinity (Hussain et al. 2018). The *OsCYP79D2* (Oryza sativa cytochrome P450 monooxygenase 79D2) and *OsCYP83A1* (Oryza sativa cytochrome P450 monooxygenase 83A1) genes catalyze early steps in GSL biosynthesis, converting methionine into aldoxime precursors, which are further processed into aliphatic GSLs crucial for stress responses. The upregulation of these genes under salt stress results in the accumulation of long-chain hydrocarbon GSLs, such as 4-methylthio-3-butenyl GSL, which offer enhanced resistance to oxidative stress and contribute to improved tolerance to high salinity (Ahmad et al. 2021). The *OsGSTF12* (Oryza sativa glutathione S-transferase F12), *OsGSTU14* (Oryza sativa glutathione S-transferase U14), and *OsGSTU5* (Oryza sativa glutathione S-transferase U5) genes encode enzymes that conjugate glutathione (GSH) to reactive intermediates, facilitating their detoxification. These GSTs help maintain redox balance under salt stress by scavenging ROS, reducing lipid peroxidation, and protecting cellular components from damage, essential for maintaining cellular homeostasis (Hussain et al. 2018). The *OsBCAT3* (*Oryza sativa* branched-chain amino acid aminotransferase 3) gene facilitates the conversion of branched-chain amino acids (BCAAs) into intermediates directed toward GSL biosynthesis. The plant’s metabolic flux shifts to produce BCAAs under salt stress, enhancing its ability to maintain osmotic balance and stabilize membranes, contributing to improved salt tolerance (Hussain et al. 2018). Additionally, the glucosinolate-myrosinase system, which breaks down GSLs into bioactive compounds like isothiocyanates, plays a vital role in activating defense pathways to improve stress tolerance. Overall, these genes *OsTGG1*, *OsTGG2*, *OsCYP79D2*, *OsCYP83A1*, *OsGSTF12*, *OsGSTU14*, *OsGSTU5*, and *OsBCAT3* work together to modulate GSL biosynthesis and breakdown in response to salt stress. Regulating enzymatic activities and influencing key metabolic pathways enhance the plant’s ability to counteract oxidative and osmotic stress, providing valuable insights for improving crop salt tolerance through genetic manipulation. It is necessary to elaborate in a deeper level of knowledge on the effects of the physiological stage, salinity tolerance, and even the responses between different glucosinolates within one scenario induced by Salt stress, especially inside the glucosinolate-myrosinase system. The effect of 40 mM salinity has been investigated in broccoli inflorescences, and results showed that the glucosinolate content considerably increased (Salehin et al. 2019). Nonetheless, this salinity escape of GLSs is attenuated by raising the Sal- level (80 mM).

## Hormonal Crosstalk in Regulating Salinity Stress Tolerance in Rice

**Fig. 5:**
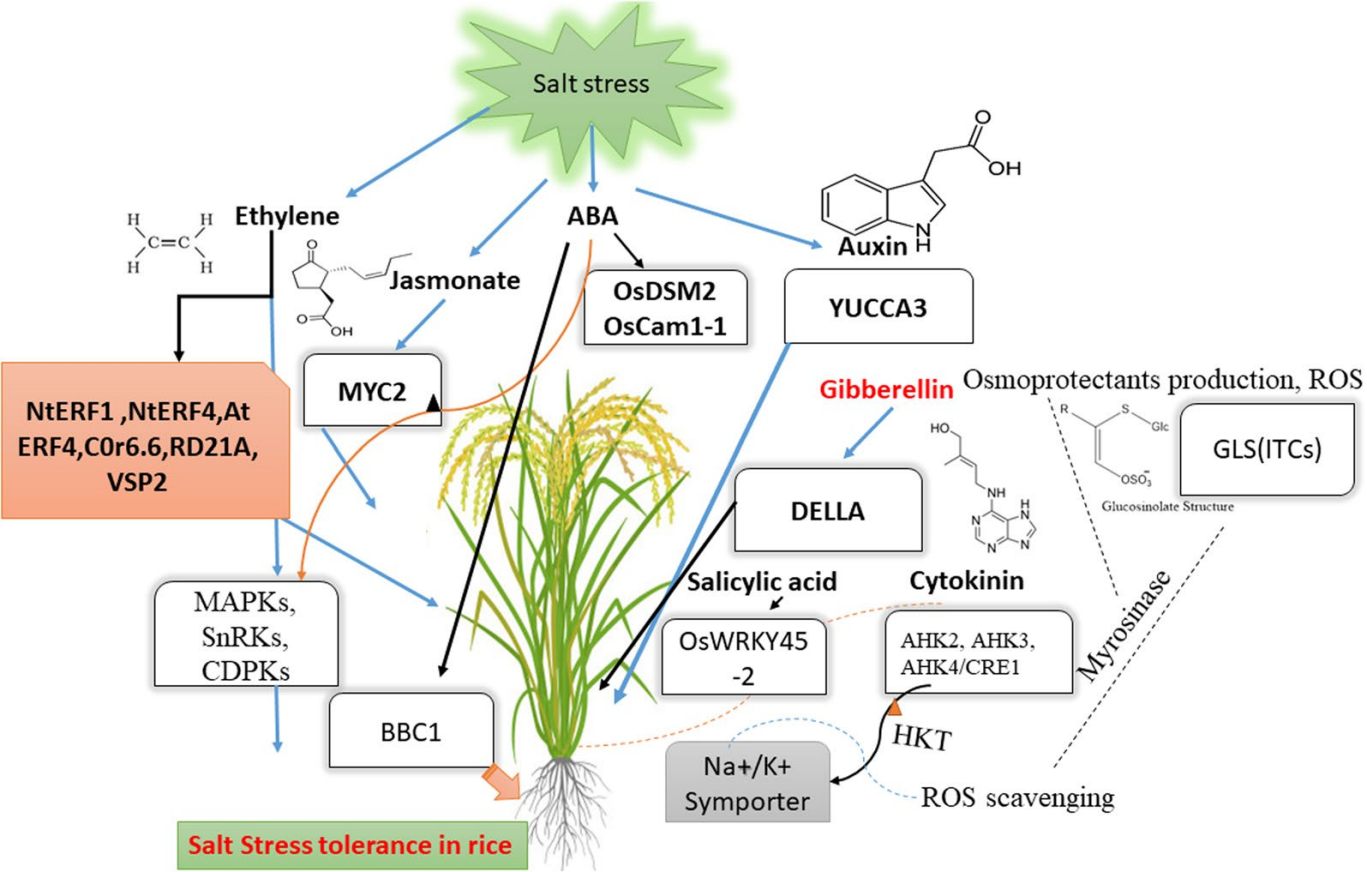
Hormonal crosstalk and glucosinolate pathway regulation under salt stress: significantly impacts plant physiology and secondary metabolism, modulating key signaling pathways and molecules. This figure illustrates the complex interplay between hormones such as auxin, abscisic acid (ABA), cytokinin, jasmonate, and ethylene in regulating salt stress tolerance in rice. It highlights the roles of specific genes, proteins, and hormones, including YUCCA3, ABA, MAPKs, CDPKs, SnRKs, OsWRKY45, NtERF1, VSP2, DELLA, and MYC2, which are integral to these stress response pathways. The up-regulation and degradation of glucosinolate biosynthesis genes such as OsTGG1, OsTGG2, OsCYP79D2, and others are also shown, revealing the intricate regulatory networks that govern the plant’s response to salt stress. The interactions between these signaling components, alongside the failure of repression and activation of specific pathways, demonstrate the plant’s adaptive mechanisms to maintain physiological balance under saline conditions

Abiotic stresses significantly impact rice production, affecting up to 70% decrease in yield (Shamsudin et al. 2016).Plant hormones play a crucial role in enhancing the ability of rice plants to acclimate to various abiotic stresses, including drought (Zhang et al. 2017; Li et al. 2019; Khan et al. 2021), salt (Misratia et al. 2015), heavy metal (Farooq et al. 2015; Verma et al. 2020), cold (Tiwari et al. 2016), heat (Seo et al. 2017) and flooding (Azra et al. 2020). The growing recognition of plant hormones in ameliorating abiotic stress tolerance mechanism in rice is discussed in this section, with further details provided in Table 1 and Fig. 5.

### Auxin in Developing Salt Stress Tolerance in Rice

Auxin is famous for regulating many aspects of plant growth and development. Thus, a differential expression during stress conditions of some auxin biosynthesis genes and transporters is responsible for rice stress tolerance (Table 1 and Fig. 1). Khan et al. (2023) explained mechanistically a rice Aux/IAA gene (*OsIAA6*), which is highly enhanced by drought stress and which, when over-expressed, contributes to drought stress resistance. (Zhang et al. 2016) also found that an auxin efflux carrier gene, *OsPIN3t*, is localized in the rice vascular tissue and is required for root and shoot development and water balance in drought-stressed rice. Khan et al. (2023) also observed that drought stress up-regulated the expression of the *OsTIR1*, *OsPIN2*, and *OsPIN5b* of rice plants but down-regulation of PINOID (*OsPID1* and *OsPIDL1*) and Crl1 (crown rootless 1) homolog in rice genome was observed in drought-stressed rice plant. External application of IAA improves the expression of genes involved in auxin signaling, including auxin receptor genes (*TIR1* and *AFB2*), transcription factors/chromatin-binding protein genes (*SPL* and *TFL2*), and the auxin response factor target gene (HAF) under drought stress (Li et al. 2019). Apart from this, RNA interference (RNAi) rice lines with low OsGRX8 transcript levels exhibit reduced root growth in the presence of auxin (1 μmol/L) as compared with wild type, indicating that IAA inducible *OsGRX8* expression may be employed profitably for the manipulation of genes related to salt and drought stresses using genetic engineering technology (Khan et al. 2023; Nicholl 2023).

In rice plants, auxin can positively regulate salt resistance. Tang et al. (2019) analyze the steady-state levels of several genes associated with auxin homeostasis in rice roots grown under salt-stress conditions. The transcription levels of *OsPIN1*a, *OsPIN1*b, *OsPIN2*, *OsPIN3*a, and *OsPIN5*b and other auxin transport genes and rice roots show higher alteration that rice has a higher ability to maintain auxin homeostasis under salt stress. In addition, Sohag et al. (2020) also found that over-expression of the α-expansin gene *OsEXPA7* significantly increases salt stress tolerance in rice. This could be due to auxin-induced cell differentiation, redox state, and enhanced levels of antioxidant machinery in response to salt stress. Additionally, for foliar application of IAA (50 μmol/L) at the flowering stage, the number of spikelets per panicle, seed-setting efficiency, and grain yield of the rice plant under salinity stress conditions may be improved because of the positive change in the antioxidant defense property.

### Role of Abscisic Acid (ABA) in Stress Signaling and Adaptation

As a signaling mediator, abscisic acid (ABA) is an essential phytohormone within the plant kingdom, which has been well-established as a pivotal orchestrator of numerous aspects and responses in the development and adaptation to myriad stress conditions under various environmental factors (El Mahi et al. 2019; Hasanuzzaman 2022). To this end, the transcription of ABA biosynthetic genes also appears to be Ca^2+^-dependent and subject to stress-induced phosphorylation events that result in signaling cascades (Ahmadizadeh et al. 2016). In addition, overexpression of ABA biosynthesis-related stress-inducible genes such as *OsDSM2* (Drought-hypersensitive mutant2) and OsCam1-1 in rice results in a striking enhancement of cellular levels of free ABA, thus improving plant growth under salty environments. *OsDSM2* and *OsCam1-1* are the genes encoding β-carotene hydrolase for ABA biosynthetic enzyme and Ca^2+^ binding calmodulin, respectively reported by Jing et al. (2016). Possible Hypothetical Model Salt stress-induced Ca^2+^ spikes may thus positively influence ABA anabolism through distinct phosphorylation events (Fig. 2).

Additionally, under drought and salt stress conditions, enhanced ABA signaling will likely upregulate the expression of salt-tolerant MAPKs (Mitogen-activated protein kinases) in rice (Zhang et al. 2016). The MAPK signaling pathway contains the sequentially acting protein kinases MAPKKK, MAPKK, and MAPK, crucial in mediating plant responses to stress (Alnayef et al. 2020). The plant hormone auxin is essential in regulating plant growth and development, including vascular tissue formation, cell elongation, organogenesis, and apical dominance, primarily by establishing auxin maxima. Overexpression of the auxin biosynthetic gene *YUCCA3* has been associated with increased auxin levels, which correlates with heightened sensitivity to salt stress (Kazan 2015; Isobe and Miyagawa 2022). Notably, severe salt stress significantly modifies root architecture by influencing auxin accumulation and distribution (Kumar et al. 2022a), indicating that alterations in the formation of auxin maxima in plant tissues are linked to diminished growth. Furthermore, under salt stress, a marked decrease in Indole-3-acetic acid (IAA) concentration has been observed in crops like rice and tomato (Ahmad et al. 2018).

### Cytokinin (CKs) in Developing Salt Stress Tolerance in Rice

Cytokinin (CK) receptors, including AHK2, AHK3, and AHK4/CRE1, are key components of the cytokinin signaling pathway, which regulates plant growth, development, and stress responses. These receptors function as histidine kinases that perceive cytokinins and initiate a phosphorelay signaling cascade, leading to the activation of type-B response regulators (RRs), which in turn regulate gene expression (Wang Bo et al. 2019; Tang et al. 2023). Under salt stress, the decline in cytokinin biosynthesis reduces signal transduction through these receptors, leading to physiological and molecular changes that influence salt tolerance. AHK2 and AHK3 are primarily involved in shoot growth regulation, while AHK4/CRE1 plays a dominant role in root development and cytokinin perception in the vasculature. The inconsistent results in stress-response studies of cytokinin receptor mutants may stem from several factors, including genetic redundancy, differences in experimental conditions, and tissue-specific roles of these receptors. For instance, some studies suggest that cytokinin signaling enhances stress tolerance by maintaining shoot growth and delaying senescence, while others indicate that reduced cytokinin signaling can improve root-to-shoot communication, enhancing stress adaptation (Wang Bo et al. 2019; Tang et al. 2023). These contrasting effects highlight the complexity of cytokinin regulation and its context-dependent role in salt stress tolerance.

### Gibberellins (GAs) in Developing Salt Stress Tolerance in Rice

Gibberellins (GAs) are crucial in regulating plant growth under abiotic stress, primarily through DELLA proteins, which inhibit growth upon stress exposure (Qin and Huang 2020). Under salt stress, endogenous bioactive GAs decline, accumulating DELLA protein and suppressing primary growth processes, such as root elongation and flowering. However, stress-induced growth constraints are alleviated in DELLA quadruple loss-of-function mutants, suggesting that DELLA proteins act as central regulators of salt stress adaptation (Kim et al. 2017). Ethylene also plays a critical role in salt stress tolerance by modulating stress-responsive gene expression. The overexpression of *NTHK1*, an ethylene receptor, enhances the expression of stress-related genes such as AtERF4, a transcription factor involved in regulating stress-adaptive responses; Cor6.6, a cold- and salt-inducible gene that protects cellular structures under osmotic stress; RD21A, a cysteine protease linked to programmed cell death and stress adaptation; and VSP2, a vegetative storage protein associated with oxidative stress resistance. Meanwhile, the downregulation of genes such as *BBC1* (a negative regulator of stress responses), Lea (a late embryogenesis-abundant protein involved in desiccation tolerance), and AtNAC2 (a transcription factor modulating root growth under salt stress) suggests that ethylene signaling fine-tunes stress responses based on environmental cues (Kim et al. 2017; Sun et al. 2022). Moreover, *Cor6.6* expression is significantly elevated in ethylene’s role in salt stress adaptation. Additionally, the expression levels of NtERF1 and NtERF4 were enhanced considerably in NTHK1-overexpressing tobacco plants compared to wild-type controls (Sun et al. 2022), reinforcing the role of ethylene signaling in modulating salt stress responses. Beyond ethylene, glucosinolates (GSLs) contribute to salt stress tolerance by enhancing osmoprotection through metabolic adjustments. Wu et al. (2021) demonstrated that the exogenous application of GSLs in rice under salt stress significantly improved salt tolerance by enhancing antioxidant enzyme activity, reducing Na⁺ accumulation, and maintaining higher K⁺/Na⁺ ratios, thus stabilizing ion homeostasis. GSLs and their derivatives, isothiocyanates (ITCs), are not only responsible for various flavors such as umami, bitterness, and astringency (Cocetta et al. 2018), but also act as signaling molecules involved in stress responses. The observed effects may be attributed to metabolic shifts aimed at adjusting turgor pressure, as the accumulation of glucosinolates in leaves with reduced water potential suggests that primary metabolism and growth are constrained under salinity stress. However, secondary metabolism, including GSL biosynthesis, remains active or is even upregulated in the adaptive response (Cheng et al. 2022). This increase in GSLs is associated with their role in osmoprotection, likely through their involvement in ROS scavenging, ion transport regulation, and stress-induced signaling pathways that mitigate the damaging effects of salt stress. These findings highlight the interconnected roles of ethylene signaling and glucosinolate metabolism in enhancing plant resilience to salinity.

## Secondary Metabolites and Calcium Signaling Under Salt Stress in Rice

Salinity stress poses significant challenges for rice (*Oryza sativa*), negatively impacting growth and development due to ion toxicity from excessive sodium ion (Na⁺) accumulation (Hussain et al. 2024). The rice response to salinity involves complex ion transport mechanisms and calcium (Ca^2^⁺) signaling pathways contributing to its tolerance (Liu et al. 2022). Under saline conditions, intracellular and extracellular Ca^2^⁺ stores mobilize to release Ca^2^⁺ into the cytosol, triggering adaptive responses (Arefian and Prasad 2022). Specific calcium channels, including cyclic nucleotide-gated (CNGCs), are crucial for maintaining ion homeostasis. For instance, *OsCNGC14*, a homolog of *AtCNGC10* from Arabidopsis, regulates Na⁺ and K⁺ transport, and mutations in *OsCNGC14* increase sensitivity to salt, resulting in elevated Na⁺ levels and disrupted Na⁺/K⁺ ratios (Ahmad et al. 2018).

Calcium-dependent protein kinases (CDPKs) are also vital for salinity stress responses in rice. For example, *OsCDPK7* activates stress-responsive genes related to ion transport and reactive oxygen species (ROS) detoxification, enhancing salt tolerance (Seifikalhor et al. 2019). Secondary metabolites, including phenolic compounds and flavonoids, protect against oxidative damage and improve resilience under salt stress. Their production is often regulated by Ca^2^⁺ signaling pathways, with Ca^2^⁺ influx during salt stress activating phenylalanine ammonia-lyase (PAL), a key enzyme in the biosynthesis of these compounds (Hao et al. 2022). Vacuolar calcium exchangers, such as *OsCAX1*, help regulate cytosolic calcium levels by sequestering excess Ca^2^⁺ into vacuoles, preventing calcium toxicity (Heinze et al. 2018). Maintaining a low Na⁺/K⁺ ratio is essential for rice survival under saline conditions. Calcium signaling influences K⁺ uptake and transport by modulating the activity of K⁺ channels (Shabala et al. 2020).

**Fig. 6:**
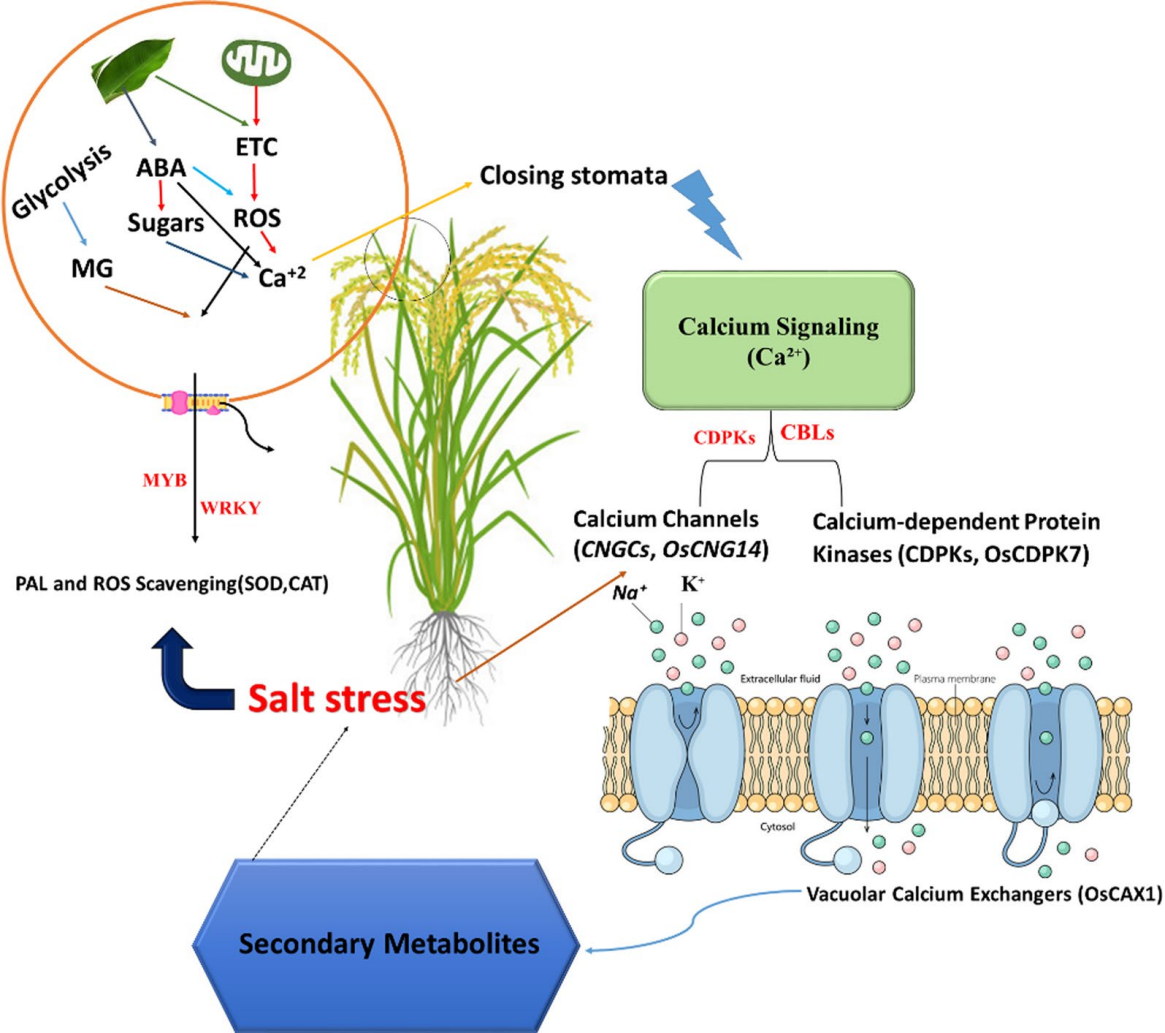
Schematic representation of calcium signaling and secondary metabolite pathways in rice under salt stress. (1) Salt stress triggers calcium signaling (Ca^2^⁺ influx), activating calcium channels (CNGCs, OsCNGC14) and calcium-dependent protein kinases (CDPKs, OsCDPK7). (2) These signals regulate ion homeostasis by modulating Na⁺/K⁺ transporters and vacuolar calcium exchangers (OsCAX1). (3) Secondary metabolites, including phenolic compounds, flavonoids, and glucosinolates, are influenced by calcium signaling and contribute to stress adaptation through ion regulation and ROS scavenging (SOD, CAT). (4) Abscisic acid (ABA) interacts with calcium signaling to regulate stomatal closure and stress-responsive gene expression. (5) Feedback loops from secondary metabolites to calcium signaling further fine-tune salt stress responses. Arrows indicate the directional flow of regulation and interactions within the pathway

The crosstalk between secondary metabolites (SMs) and calcium (Ca^2^⁺) is crucial in enhancing rice resilience to salt stress. The influx of Ca^2^⁺ through plasma membrane channels is an essential trigger for calcium-dependent signaling pathways (Fig. 6). This increase in cytosolic Ca^2^⁺ activates calcium sensors, such as calmodulins and calcineurin B-like proteins (CBLs), which regulate stress-responsive genes (Cocetta et al. 2018). These sensors modulate ionic homeostasis by regulating Na⁺/K⁺ transporters, protecting plants from ionic toxicity induced by salt stress (Kazan 2015). Calcium signaling exhibits a complex interplay with secondary metabolites that enhance stress tolerance through multiple layers of protection.

Secondary metabolites like flavonoids, phenolics, alkaloids, and glucosinolates are essential for alleviating damage caused by abiotic stresses such as salinity. They primarily protect the plant from oxidative damage induced by excessive ROS production during salt stress (Nicolas-Espinosa et al. 2023). Ca^2^⁺ signaling modulates ROS levels by activating ROS-scavenging enzymes, including superoxide dismutase (SOD) and catalase (CAT), which work alongside SMs. This calcium-SM interaction helps maintain cellular integrity under salt stress. Moreover, Ca^2^⁺ signaling directly influences the biosynthesis of SMs. Calcium-binding proteins, like CBLs and CDPKs, regulate key transcription factors such as MYB and WRKY, which control the expression of genes involved in SM biosynthesis (Cheng et al. 2022). For example, under salt stress, glucosinolates accumulate, providing defense by acting as antioxidants and playing roles in Osmo protection and signaling. SMs like phenolics reinforce cell walls, enhance mechanical strength, and reduce the permeability of harmful ions such as Na⁺ (Ma et al. 2016).

In turn, secondary metabolites (SMs) exert feedback control over calcium signaling, modulating the activity of calcium channels and transporters. For instance, polyamines, such as spermidine and spermine, stabilize cell membranes, preventing excessive Na⁺ influx under salt stress, thus mitigating ionic toxicity (Bo et al. 2019). These polyamines interact with calcium channels, reducing the entry of Na⁺ ions that directly impact the cell’s calcium signaling network by preventing the overload of calcium ions that would otherwise disrupt cellular homeostasis. Additionally, certain phenolic compounds, such as quercetin, influence calcium transporters like Ca^2⁺^-ATPase, ensuring proper calcium homeostasis during stress. These secondary metabolites help maintain the optimal calcium levels for signaling without causing cellular damage. The interplay between calcium signaling and SMs is crucial for regulating hormonal pathways during salt stress, particularly the modulation of abscisic acid (ABA) biosynthesis and signaling. Calcium-dependent protein kinases (CDPKs) activate ABA-responsive elements essential for stress-induced stomatal closure and ion homeostasis. Flavonoids, a class of SMs, modulate calcium-dependent ABA signaling by enhancing the sensitivity of ABA receptors and influencing the expression of ABA-responsive genes (Liu et al. 2020). This action is especially evident in regulating stomatal movements, where flavonoids help fine-tune calcium ion flux, promoting ABA’s ability to regulate stomatal closure, thus preventing excessive water loss during salt stress.

Moreover, calcium signaling acts as a second messenger, stimulating the biosynthesis of SMs and enhancing their functional activities in mitigating salt-induced damage. The increased production of SMs like flavonoids and phenolic acids stabilizes cell membranes, improving osmotic balance and reducing oxidative stress by scavenging ROS. This alleviates the detrimental effects of salt stress and enhances the plant’s overall tolerance. Calcium signaling also activates stress-related transcription factors like NAC and AP2/ERF families, which are crucial for coordinating the stress response at the gene expression level. These transcription factors work synergistically with SMs to regulate pathways associated with ion transport, osmotic adjustment, and ROS scavenging, which are essential for combating salt stress. This integrated defense mechanism, where calcium and SMs work in concert, greatly enhances salt tolerance in rice. For example, flavonoids contribute to ion homeostasis by interacting with calcium channels and transporters, preventing the excessive influx of Na⁺ ions while promoting calcium’s beneficial role in cellular signaling. In summary, calcium signaling not only activates the synthesis of SMs but also amplifies their functional role in regulating gene expression, ion transport, osmotic protection, and ROS scavenging, collectively improving the plant’s resilience against salt-induced stress (Tiwari et al. 2016).

## SMs Modulate Gene Expression Under Salt Stress

To elucidate the roles of pivotal genes in rice roots, RNA-seq datasets from the Sequence Read Archive (SRA) were meticulously searched, retrieved, and analyzed (Bundó et al. 2022). This QTL-based study aimed to delineate the global transcriptional alterations in rice subjected to salt stress by associating gene expression in control samples and samples treated with 80 mM NaCl for 24 h, each with three biological replicates. Unsupervised hierarchical clustering analysis identified nine significantly up-regulated genes and six down-regulated genes under salt stress conditions relative to the control (Table 2).

**Table 2:** Differential expression and regulation of rice genes under salt stress conditions

Gene name	Fold change value	Log2FC value	*P*-value	Up/downregulated
*OsCPK12*	2.699052133	1.432452843	0.019780836	Up-regulated
*OsLOL5*	1.623595506	0.699192252	0.022415271	Up-regulated
*OsMAPK44*	1.631455399	0.706159547	0.159223088	Up-regulated
*OsJRL40*	1.5	0.584962501	0.507388351	Up-regulated
*OsSAPK4*	2.054306089	1.038651157	0.037957061	Up-regulated
*OsKAT1*	1.333333333	0.415037499	0.422649731	Up-regulated
*OsTPS8*	1.360655738	0.444302094	0.672425311	Up-regulated
*OsBADH1*	1.090909091	0.125530882	0.477767032	Up-regulated
*OsMYB91*	0.253164557	− 1.981852653	0.093807834	Downregulated
*OsVP1*	0.673913043	− 0.569365646	0.497481092	Down-regulated
*OsNHX1*	2.810964083	1.49106502	0.29631443	Up-regulated
*OsHKT1;1*	0.771228771	− 0.374769222	0.525213945	Down-regulated
*OsHKT1;4*	0.9	− 0.152003093	0.825922344	Down-regulated
*OsHKT1;5*	0.5	− 1	0.225403331	Down-regulated
*OsHAK5*	0.777456647	− 0.363165865	0.489954366	Down-regulated

**Fig. 7:**
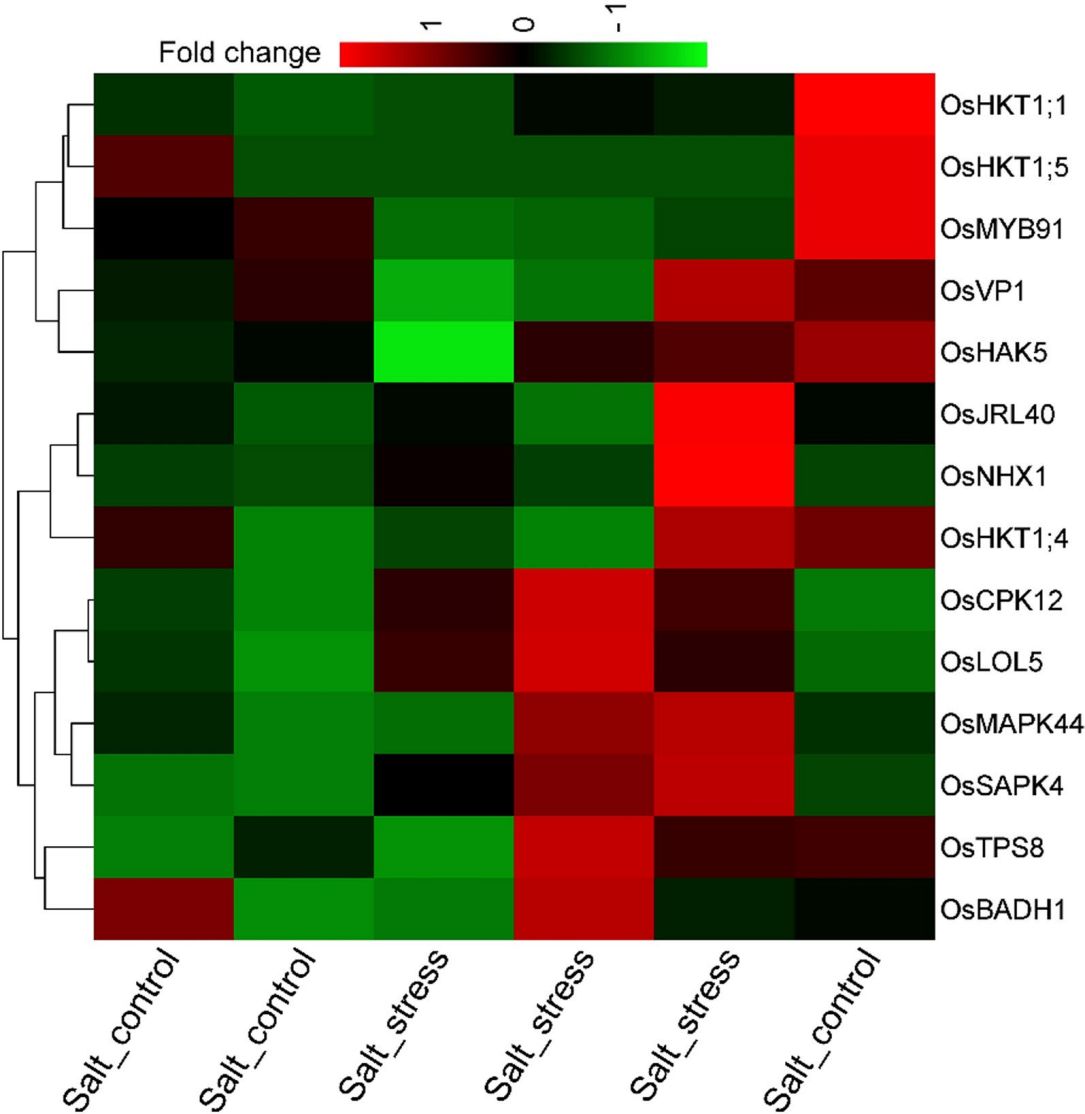
Differential gene expression patterns in rice under control and salt stress conditions. The heatmap, generated using iDEP-v2.01 (http://bioinformatics.sdstate.edu/idep/), displays the fold change in gene expression based on transcriptomic data from SRA (Accession: GSE167342). Red indicates genes with upregulated expression under salt stress, while green represents downregulated genes, highlighting the transcriptional response to salinity stress

Notably, all critical genes exhibited higher expression levels under salt stress than controls (Fig. 7). While the analysis was conducted on rice roots under salt stress, it is essential to note that these findings may reflect the responses of distinct rice cultivars, each potentially harboring specific genetic variants. These genetic differences could explain the varying functions of the vital genes across cultivars. A comparative analysis across multiple rice cultivars would be necessary to explore this further to determine how specific genetic variations influence the transcriptional response to salt stress. These results suggest that the roles of the viral genes in rice stress response may be cultivar-dependent, warranting further investigation into their function across different genetic backgrounds.

### *Protein–Protein Interaction (PPI*) *Under Salt Stress*

**Fig. 8:**
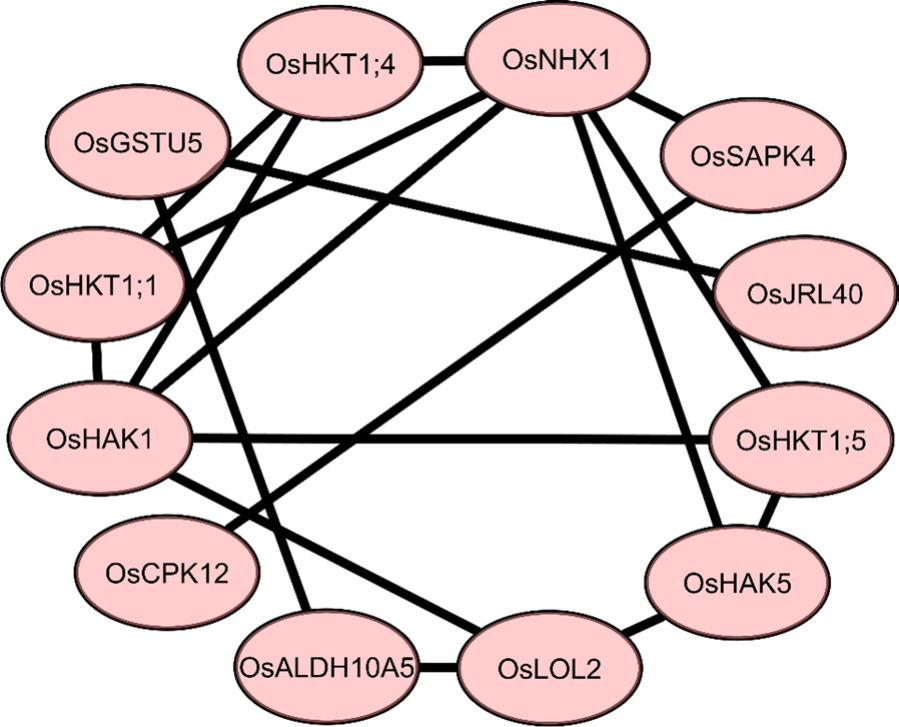
An investigation of the protein–protein interaction (PPI) network reveals the interactions among proteins involved in salt stress (SSF). The network’s nodes represent the proteins, while the edges represent the interactions. The connections between nodes indicate the level of interaction between SSF proteins based on their degree value. The sources and locations of conjugation show the origin and targets of SSF proteins within the network. A higher number of connections at a node signifies increased interactions between SSF proteins and that specific SSF

The close interactions between *OsGSTU5*, *OsHKT1;1*, *OsHAK5*, *OsHKT1;4*, and *OsCPK12* under salt stress are crucial for maintaining ion homeostasis and cellular integrity. *OsGSTU5* (a glutathione S-transferase) plays a pivotal role in detoxifying reactive oxygen species (ROS) and protecting cells from oxidative damage. *OsHKT1;1* and *OsHKT1;4* are involved in sodium ion (Na⁺) transport and homeostasis, and their interaction with *OsHAK5* (a high-affinity potassium transporter) ensures balanced ion uptake and storage, critical for plant survival under salt stress. *OsCPK12* (a calcium-dependent protein kinase) acts as a key mediator in stress signaling and activates downstream responses to mitigate the effects of high salinity (Fig. 8). The close interaction between these proteins reflects their coordinated roles in adjusting ion flux, managing oxidative stress, and triggering appropriate physiological responses to salinity. On the other hand, the more distant interactions between *OsJRL40*, *OsSAPK4*, and *OsHAK5* may reflect the involvement of these proteins in different pathways, such as stress perception, signaling, and transcriptional regulation, which are not directly linked but still crucial for the plant’s response to salt stress (Bo et al. 2019).

### Phylogenetic Relationship Analysis

**Fig. 9:**
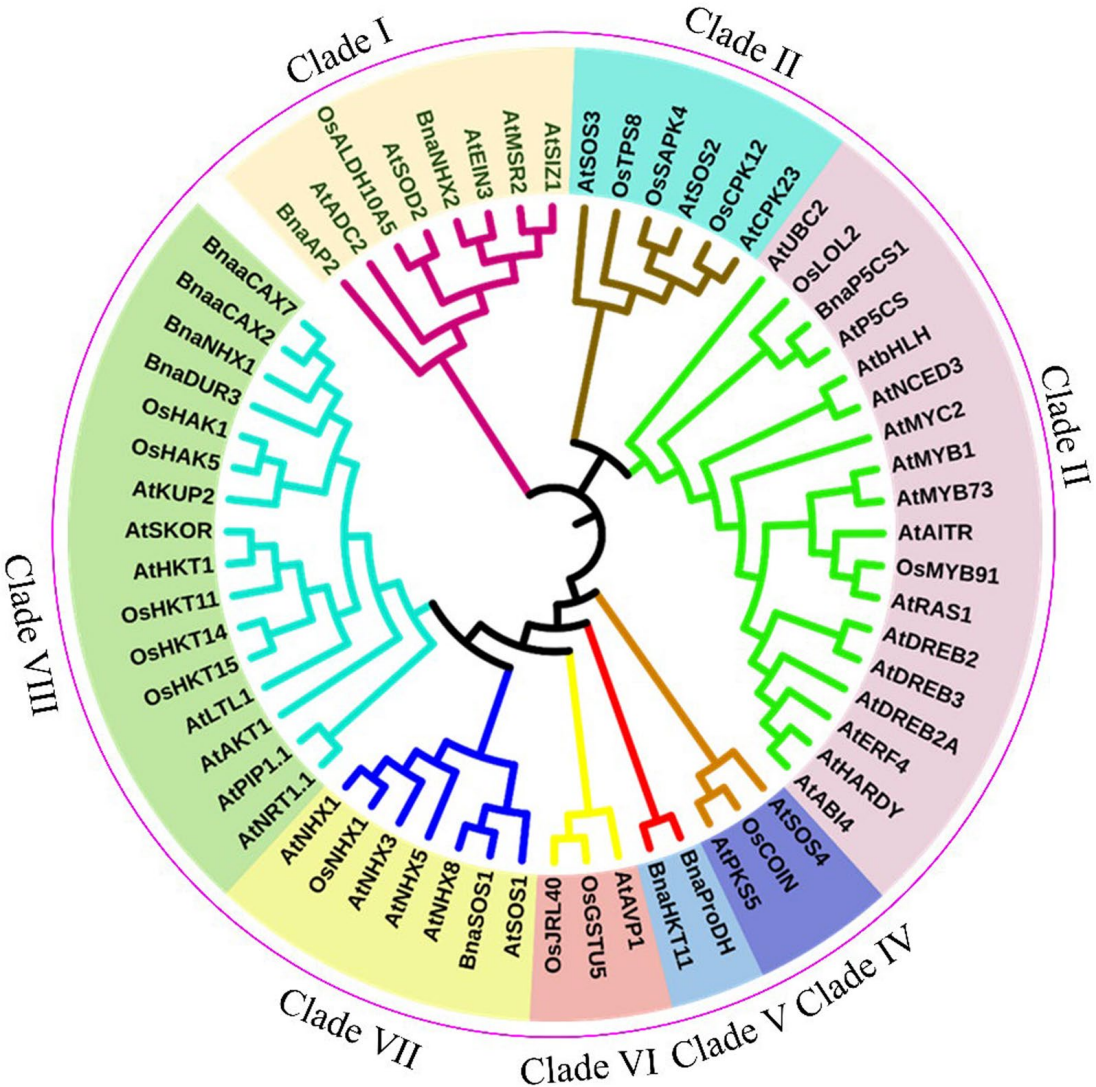
The expression changes of salt-tolerance-related genes in Na^+^/ K^+^ ion homeostasis (upper right), osmotic adjustment (upper left), ROS scavenging (lower left), and nutritional balance (bottom right) under long-term high salinity conditions. The central yellow Stripe shows the functional genes encode proteins that protect membranes and macromolecules against stress. Protective protein-coding genes are underlined in green (apex) and pink. They represent a peripheral layer surrounding the core of functional or enzyme-coding genes that protect plants from diverse stresses by regulating their expression

An unrooted phylogenetic tree was constructed using multiple protein sequence alignments from the rice, brassica, and Arabidopsis families to investigate the evolutionary relationships and classifications of critical genes involved in salt stress. These proteins were categorized into eight subfamilies (Clade I to Clade VIII). Subfamily Clade III, comprising 18 members, was identified as the largest group, whereas Subfamily Clade V, with only two brassica proteins, was among the smallest. Subfamilies Clade IV and Clade VI each consisted of three proteins from rice and Arabidopsis, respectively (Fig. 9).

Several genes regulate the expression of brassica and Arabidopsis families, hence affecting rice salt tolerance. These include specific protein-based genes such as *OsMYB91*, *OsHKT1*, *OsHKT1;5*, *OsLOL2*, and the sucrose no fermenting 1-related protein kinase2 (HAK1) HAK4 have been summarized in Fig. 8 and Table 2. These proteins directly stimulate the production of K^+^/Na ^+^ transporters, leading to an amended K^+^/Na ^+^ ratio and a good effect on salt tolerance (Cui et al. 2018; Patishtan et al. 2018; Shofiqul et al. 2019). The interactions of myristoylated Ca^2+^ binding proteins are seen in all clades. Clade II phosphorylates Clade VII, which activates the Na^+^/H^+^ antiporter OsSOS1 and enhances rice’s ability to tolerate salt stress (Shofiqul et al. 2019). The MYB transcription (MYB) *OsMYB91* has a detrimental effect on salt tolerance as it enhances the absorption of sodium into cells, resulting in a decrease in the ratio of potassium (K^+^) to sodium (Na^+^) ions (Shofiqul et al. 2019) (Table 3; Fig. 8). MAPK (Mitogen-Activated Protein Kinase) proteins are part of a signaling cascade that transmits stress signals from the cell surface to the nucleus, where they can activate or suppress the expression of targeted genes. The findings above indicate that regulating Na^+^/K^+^ balance during salt stress could be a viable approach to enhance salt tolerance in rice.

**Table 3:** Essential genes associated with salt stress tolerance in rice

Gene Name	Function	Salt stress mechanism	Interaction with other components	Impact on rice growth/development	References
*OsCPK12*	Increases tolerance to elevated salt concentrations by decreasing ROS accumulation	ROS scavenging, stress signaling	Involves interaction with other ion transporters and antioxidant enzymes	Improves tolerance by reducing oxidative stress in cells	Xu et al. (2018); Cheng et al. (2022)
*OsLOL5*	Improves the removal of ROS and enhances salt stress resilience	ROS detoxification, cell signaling	Interacts with other stress-responsive genes and antioxidant enzymes	Increases tolerance by improving oxidative stress defense	Bundó et al. (2022)
*OsMAPK44*	Contributes to ion homeostasis in response to high salinity	Ion balance regulation under salt stress	Interacts with ion channels and transporters	Enhances ion homeostasis and improves salt tolerance	Ayadi et al. (2022)
*OsJRL40*	Increases antioxidant enzyme activity and balances Na^+^/K^+^ ion homeostasis under salinity stress	Antioxidant enzyme activation, ion homeostasis regulation	Works alongside Na^+^/K^+^ transporters and transcription factors	Boosts salt tolerance and root growth	Hasanuzzaman (2022)
*OsSAPK4*	Regulates ion homeostasis during rice growth under saline conditions	Ion balance, stress response signaling	Interacts with other signaling pathways and ion transporters	Promotes growth under salt stress by maintaining ion balance	Austen et al. (2019)
*OsKAT1*	Enhance salt tolerance by increasing potassium uptake and reducing Na^+^ accumulation	K^+^ uptake, Na^+^ exclusion	Works with other potassium transporters and ROS regulators	Improves salt tolerance, especially under high salinity	Isobe and Miyagawa (2022)
*OsTPS8*	Regulates soluble sugars and ABA signaling for salt stress tolerance	Soluble sugar regulation, ABA signaling	Involves ABA-dependent pathways and osmotic stress regulators	Improves osmotic adjustment and stress response	(Kumar et al. (2022b)
*OsBADH1*	Promotes osmoprotectant production for better tolerance to high salinity	Osmoprotectant synthesis, stress mitigation	Works with other osmoregulatory genes	Enhance plant survival under salt stress by promoting osmotic balance	Kumar et al. (2022a)
*OsMYB91*	Regulate development and salt stress tolerance	Gene regulation, stress tolerance	Interacts with salt-responsive genes and transcription factors	Aids in growth and adaptation under saline conditions	Pereira et al. (2023)
*OsVP1 & OsNHX1*	Reduces Na^+^ accumulation, enhances salt tolerance, and improves root biomass	Na^+^ homeostasis, ion transport	Interacts with Na^+^/K^+^ transporters and growth regulators	Enhances root growth and photosynthesis under salt stress	Bundó et al. (2022)
*OsHKT1;1, OsHKT1;4, OsHKT1;5*	Reduces Na^+^ buildup in shoots, enhancing salt tolerance	Na^+^ transport, stress tolerance	Interacts with Na^+^/K^+^ transporters and stress-responsive genes	Enhances tolerance by controlling Na + movement during salt stress	Wang et al. (2015)
*OsHAK5*	Helps maintain ion balance, improving tolerance to high salt levels	K^+^ uptake, Na^+^ exclusion	Works with K^+^ and Na^+^ Transporters	Improves salt tolerance by enhancing potassium uptake and Na^+^ exclusion	Wang et al. (2015)

## Conclusion

Secondary metabolites (SMs) are crucial in enhancing plant resilience to salt stress by regulating various physiological processes such as osmotic balance, ion homeostasis, and oxidative stress management. Their interaction with signaling pathways, including abscisic acid (ABA) and mitogen-activated protein kinases (MAPKs), helps mitigate cellular damage under saline conditions. Recent studies have identified key transcription factors like MYB and WRKY involved in regulating SM biosynthesis, and Meta-QTL analysis has pinpointed crucial loci associated with increased SM production in salt-stressed environments. These findings open new avenues for breeding salt-tolerant crops by targeting specific metabolic pathways.

## Future Perspectives

- Investigate the regulatory networks governing SM biosynthesis under salt stress, focusing on genetic and epigenetic factors.
- Explore the role of transcription factors (MYB, WRKY) in regulating SM pathways during salt stress.
- Integrate multi-omics approaches (Transcriptomics, Metabolomics, Proteomics) to understand better how SMs interact with broader stress response mechanisms.
- Advanced breeding technologies such as CRISPR and genomic selection can enhance SM production in crops, aiming for improved salt tolerance.
- Conduct field-based studies to assess the practical impact of SMs on crop performance in saline environments.
- Focus on metabolic engineering strategies to optimize SM pathways for sustainable agriculture, particularly in salt-affected regions.
- Investigate using gene cassettes for metabolic pathways and secondary metabolites to enhance crop immunity against environmental stresses, including salinity.

## Data Availability

No datasets were generated or analysed during the current study.
